# Egg disinfection improves larval survival and shapes the microbial community in snubnose pompano (*Trachinotus blochii*)

**DOI:** 10.1038/s41598-026-35646-8

**Published:** 2026-01-19

**Authors:** T. G. Sumithra, S. R. Krupesha Sharma, S. Gayathri, Ambarish P. Gop, K. S. Shravana, Amritha Jagannivasan, Anusree V. Nair, K. S. Sudarsan, B. Santhosh, Sanal Ebeneezar, A. Gopalakrishnan

**Affiliations:** 1 ICAR-Central Marine Fisheries Research Institute (CMFRI), Ernakulam North PO, Kochi, 682018 Kerala India; 2 Cochin University of Science and Technology, Kochi, 682022 Kerala India; 3 Department of Plant Biotechnology, College of Agriculture, Vellayani, Thiruvananthapuram, 695522 Kerala India

**Keywords:** Egg disinfection, Larval survival, 16S rRNA sequencing, Aquaculture microbiome, Host-microbe interaction, Biotechnology, Microbiology

## Abstract

Early microbial colonization is crucial for immunity and survival in aquatic animals. This study evaluated the impact of egg disinfection on microbial colonization and larval performance in *Trachinotus blochii*, a high-value mariculture fish. Optimal egg disinfection protocols were initially identified as 20 ppm iodophor for 10 min, 400 ppm H₂O₂ for 10 min, and 40 ppm glutaraldehyde for 5 min to improve hatchability. Sequential analyses included 16S rRNA amplicon sequencing of larval microbiota at 10-days post hatching (DPH) and assessment of survival and antioxidant status till 25 DPH. Disinfection significantly enhanced hatchability (up to 90.88 ± 2% with 40 ppm glutaraldehyde), larval survival (up to 34.80 ± 1.1% in glutaraldehyde and 31.18 ± 1.5% in H₂O₂), and catalase activity. Notably, egg disinfection reshaped the larval microbiota, enriching microbial diversity measures and beneficial bacterial taxa, such as *Hyphomonadaceae*, *Halieaceae*,* Nannocystaceae*, and *Alteromonadaceae*. Improved survival correlated with enhanced taxonomic and functional metagenomic diversity, lower *Proteobacteria*: *Bacteroidota* ratio and higher combined proportions of *Fusobacteriota*, *Firmicutes*, and *Bacteroidota* relative to *Proteobacteria*. The findings suggest that egg disinfection acts as a microbiota programming strategy to promote larval health, offering a practical approach to enhance sustainability in *T. blochii* aquaculture.

**Supplementary Information:**

The online version contains supplementary material available at 10.1038/s41598-026-35646-8.

## Introduction

Aquaculture is the world’s fastest-growing sector in food production, emerging as a key solution to food and nutrition security. Sustainable aquaculture requires efficient, eco-friendly hatchery practices that can enhance larval survival. Despite continuous advancements in marine fish larviculture technologies, poor larval survival remains a major bottleneck in hatchery operations^1^. Increasing evidence suggests that sustainable microbial management strategies have considerable potential to enhance larval quality and survival rates. Environmental conditions and microbial exposures during the early developmental stages of aquatic animals can exert profound and lasting effects on their microbiota, metabolism, and physiology^2^. Among the different early developmental stages, the egg phase represents a critical window during which microbial colonization can significantly influence larval health, physiological resilience, and long-term survival^3^. Recent studies have shown a strong correlation between the egg-associated microbiota and the climax microbial community structure of larvae^4^. Sullam et al.^5^ demonstrated the substantial contribution of egg microbiota to the gut microbiota in succeeding fish stages.

Traditionally, egg disinfection has been employed as a biosecurity measure to prevent vertical transmission of pathogens. However, it is now being re-evaluated as a potential tool for metabolic programming and improving larval performance. Emerging insights suggest that such early microbial interventions may go beyond pathogen control, potentially modulating microbiota assembly and host physiology in ways that influence long-term fitness^2^. Commonly used disinfectants for fish eggs include hydrogen peroxide (H₂O₂), glutaraldehyde, and iodophor, which help reduce the risk of transmitting viruses and bacteria from broodstock to offspring^6,7^. Notably, egg treatment with H₂O₂ was reported to enhance hatching success in certain fish species^7^. Moreover, exposure of eggs to H₂O₂ before hatching has been found to influence antioxidant capacity in *Cyprinus carpio*^8^ and *Micropterus salmoides*^9^, antioxidant defences, immunity, and lipid metabolism in *Misgurnus anguillicaudatus*^2^, and the immune response in *C. carpio*^10^. Accordingly, understanding how egg disinfection protocols alter these early microbial interactions and downstream physiological responses can be critical for developing more refined and effective hatchery practices. Despite these, there is a lack of comparable egg disinfection studies on tropical marine fish species and disinfectants other than H₂O₂. Furthermore, optimal disinfectant concentrations and exposure durations can vary significantly depending on the species and environmental conditions, emphasizing the need for species-specific research under relevant culture conditions^6^. Importantly, despite the widespread application of egg disinfection protocols in fish hatcheries, limited research has evaluated their broader impacts on microbial community dynamics of later life stages.

In this context, the present study provides novel insights into simple, scalable egg disinfection protocols that can strategically program larval microbiota, increasing post-hatch survival. Initially, the tolerable concentration and exposure period of three widely used disinfectants, H_2_O_2_, glutaraldehyde, and iodophor for egg disinfection in a high-value tropical marine teleost, *Trachinotus blochii* (snubnose pompano), were determined. Afterwards, we examined how disinfection before hatching influences larval survival, microbiota composition, and antioxidant capacity. By linking microbial modulation to physiological outcomes, this study also aims to identify microbial indicators of improved larval quality and survival. Briefly, this study links egg disinfection, microbial programming, and larval viability, offering novel insights into microbiome-based strategies to enhance hatchery success in marine finfish aquaculture. The findings demonstrate that pre-hatch egg disinfection can serve as a strategic intervention to improve larval robustness and overall hatchery performance of *T. blochii*, supporting cleaner and more sustainable aquaculture systems.

## Materials and methods

### Animals used

Animals maintained in the national brood bank facility of snubnose pompano at the Vizhinjam Regional Centre of ICAR- Central Marine Fisheries Research Institute (ICAR-CMFRI), India, were used. The animals were induced to spawn by the established protocols of the facility^1^, and fertilized eggs were collected for experiments.

All the methods were carried out in accordance with relevant guidelines and regulations. The experiments followed the ARRIVE guidelines^11^, the UK Animals (Scientific Procedures) Act^12^ and the EU Directive 2010/63/EU for animal experimentation^13^. The protocols for handling live animals were approved by ICAR-CMFRI, Kochi, India (BT/AAQ/3/SP28267/2018).

### Experimental design

**Fig. 1:**
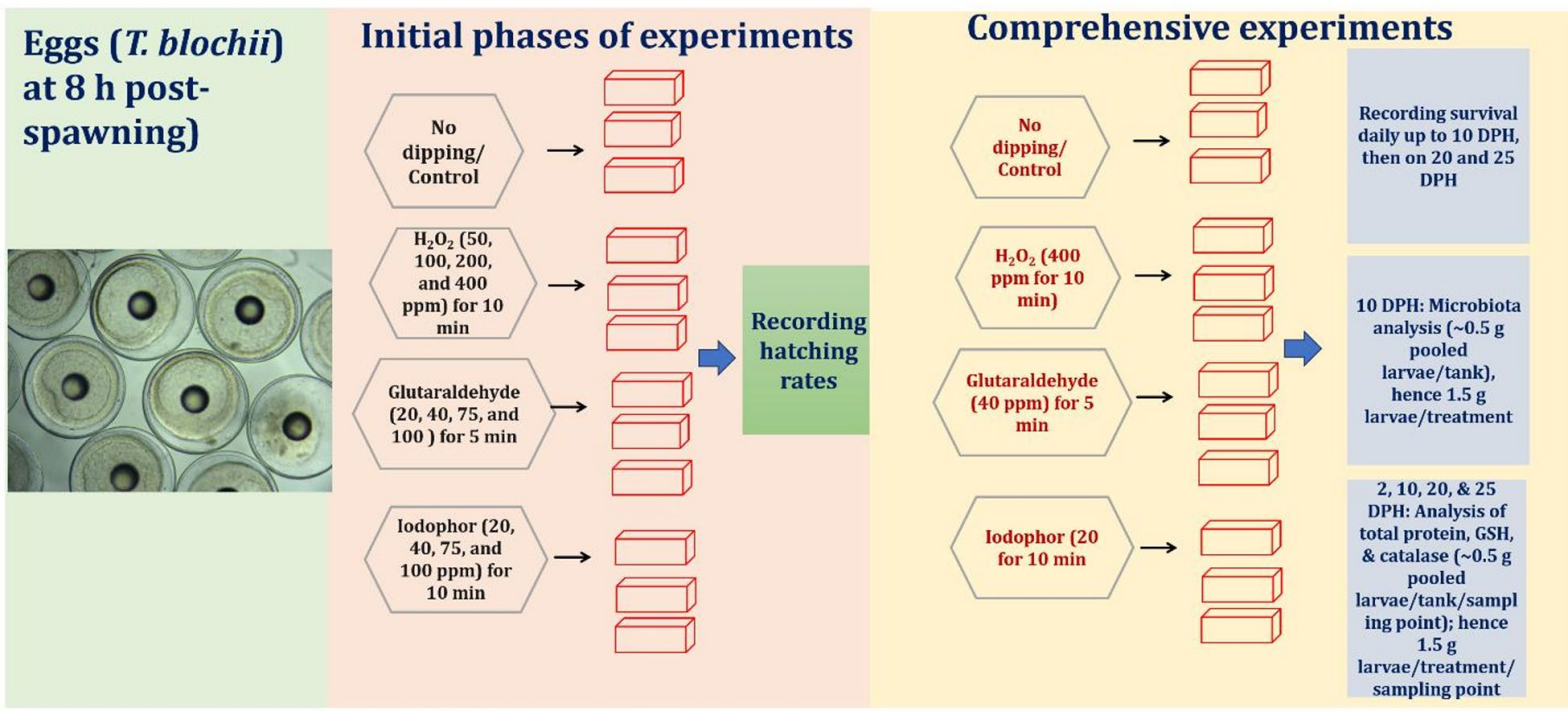
A schematic of the overall experimental design in the present study. DPH: Days post hatching; H_2_O_2_: Hydrogen peroxide; ppm: Parts per million; min: Minutes; GSH: Reduced glutathione.

The collected floating eggs from the broodstock tank at the optic vesicle stage of embryonic development (8 h post-spawning) were randomly counted in triplicate using subsamples of 5–10 ml of seawater containing eggs, and the total number of eggs was estimated by multiplying the average egg count in the samples with the total volume of seawater used. The initial phases of experiments were conducted with varying concentrations of 30% H_2_O_2_ solution (EMPLURA^®^, Sigma), 50% glutaraldehyde in water (Sigma, USA), and iodophor with 1.6 to 1.7% available iodine (Qualigens; pH of 0.1% solution: 3 to 4). Egg hatching rates [(numbers of larvae/number of eggs stocked) *100] were only recorded. The comprehensive experiments were done using the selected concentration of H_2_O_2_, iodophor, and glutaraldehyde. Here, the larval survival rates, influences on microbiota, and antioxidant parameters were recorded. The experimental phases are shown in Fig. 1. During all experiments, each treatment and control (eggs without any treatment) group was maintained in triplicate tanks (*N* = 3). After the treatment, the eggs were stocked at a density of 10,000 per 5-tonne Fibre Reinforced Plastic (FRP) larval rearing tank containing *Nanochloropsis oceanica* and *Isochrysis galbana*, microalgae rich in essential nutrients, especially polyunsaturated fatty acids, proteins, and vitamins^14,15^. Rearing followed standard practices of the national brood bank facility for snubnose pompano at the Vizhinjam Regional Centre of ICAR-CMFRI, India^1^. Water quality was maintained at a temperature of 28 ± 1 °C, salinity of 34 ± 1 ppt, and pH of 8.2 ± 0.7. Ammonia (< 0.3 mg/L) and nitrite (< 0.04 mg/L) levels were monitored daily.

### Initial phase of the experiment

Iodophor solutions containing varied concentrations of active iodine, viz. 10, 20, 50, and 100 ppm, were prepared in 4 L seawater in buckets, and the pH of each solution was adjusted to 8 using 1 N sodium bicarbonate. The collected eggs were dipped in each dilution for 10 min and stocked. Similarly, varied concentrations of H_2_O_2_, viz. 50, 100, 200, and 400 ppm, were prepared in 4 L of seawater in buckets, and the pH of each disinfectant treatment solution was adjusted to 8 using sodium bicarbonate. The collected eggs were dipped in each dilution for 10 min and transferred for stocking. Likewise, varied concentrations of glutaraldehyde, viz. 20, 40, 75, and 100 ppm, were prepared in 4 L of seawater in buckets, and the pH of each solution was adjusted to 8 using sodium bicarbonate. The collected eggs were dipped in each dilution for 5 min. Immediately after each treatment, the eggs were rinsed in seawater and stocked in triplicate tanks.

### Comprehensive experiment

The comprehensive experiment was done using the selected concentration-time combination of H_2_O_2_, iodophor, and glutaraldehyde, based on the results of the initial phase. The larval survival rates (daily survival up to 10, 20 and 25 DPH) were estimated by the formula: (Numbers of live larvae in each tank on observation day/numbers of hatched larvae in each tank on 2 DPH) * 100. The samplings for microbiota analyses were done on the 10th day of stocking after recording the larval survival rates. The observation of larval malformations was carried out through routine visual and microscopic examination using a stereo zoom microscope at various developmental stages. For microbiota analysis, approximately 0.5 g (wet weight) of larvae were randomly collected from each experimental tank (triplicate samples for each treatment) at 10 DPH and processed. We selected 10 DPH for microbiota analysis since this stage represents a critical window in larval development, when feeding is fully established^4^, and significant differences in survival among treatments (in the present study) were observed. Three independent tanks were maintained for each treatment, and one pooled sample (0.5 g) was collected from each tank, resulting in three biological replicates per treatment (1.5 g/ treatment). This design ensured that replicates represented independent tank-level units, and randomisation was incorporated during larval pooling. The larval pools were washed with sterile seawater and transferred to a cryotube containing 3 ml absolute ethanol and rapidly frozen at -20 °C^4^.

For profiling the total protein content and catalase activity, 0.5 g larval samples/ tank/ sampling point (on 2, 10, 20, and 25 DPH) were collected in 2 ml of PBS (pH = 7.4) and preserved at -20 °C till assay. For quantifying reduced glutathione content, fresh larval samples (~ 0.5 g larval samples/ tank/ sampling point) were directly used for the assay without preservation^16^.

### Profiling the antioxidant parameters

The preserved larval samples were homogenized in the same buffer (PBS) after thawing at 4 °C and centrifuged at 10,000 rpm for 20 min at 4 °C. The supernatant was used for the assays.

#### Measurement of protein content

Total protein of larval homogenate was measured using Lowry’s method. Briefly, 100 µl of 1:1 diluted homogenate in PBS (pH 7.4) was treated with 500 µl of alkaline copper sulphate reagent and kept for incubation at room temperature for 10 min. Then 50 µl of Folin-Ciocalteau reagent (Himedia, India) was added and further incubated at 37 °C for 30 min in the dark. The optical density (OD) was read at 660 nm. Total protein content was estimated in mg per ml larval homogenate from the standard curve prepared by using serial dilutions of bovine serum albumin standard solution.

#### Assay for catalase activity

Catalase activity in the larval homogenate was measured following the previous protocol^16^. Briefly, 1 ml of 1: 10 diluted sample (in 0.01 M potassium phosphate buffer, pH 7.2) was reacted with 320 µl of  30% H_2_O_2_ for 3 min at 37 °C. Around 1 ml of this mixture was combined with 2 ml of dichromate/ glacial acetic acid (1:3) solution to form an unstable blue-coloured complex. The solution was boiled to develop a stable green-coloured chromic acetate, which was cooled to room temperature. The OD was read at 570 nm. The catalase activity was estimated in units per mg of larval protein content, where one unit is the enzyme decomposing 1 µM H_2_O_2_ per min at 37 °C^16^.

#### Measurement of reduced glutathione content (GSH)

The 5, 5-dithio-bis (2-nitrobenzoic acid) method (DNTB) was followed to estimate the GSH content^16^. Briefly, 100 µl of the sample was mixed with 100 µl of 5% sulphosalicylic acid and incubated for one hour at 4 °C. The mixture was centrifuged, and 50 µL of the supernatant was mixed with 275 µL PPB (0.1 M, pH 7.4). Around 50 µL DTNB (Ellman’s reagent) was added to this mixture, and OD was estimated immediately at 412 nm. The GSH content was expressed as µg per mg of larval protein content.

### Profiling of larval microbiota

The preserved larval samples were processed as per the previous protocol to minimize host cell contamination^17^. The metagenomic DNA was then extracted using the QIAamp DNA Microbiome Kit (Qiagen). The integrity of DNA was evaluated on 1% agarose gel. Quantification and purity of DNA were checked using Nanodrop 2000 (Thermofisher Scientific, Massachusetts, USA). The DNA samples were preserved at -20℃ until sequencing.

#### Preparation of metagenomic library, cluster generation and sequencing

The V3-V4 region of the 16S rRNA gene was amplified with the forward primer, 341 F (CCTACGGGNGGCWGCAG) and reverse primer, 805R (GACTACHVGGGTATCTAATCC), utilising the KAPA HiFi HotStart Ready Mix (Roche, Switzerland). The sequencing was performed at Nucleome Informatics Private Limited (Hyderabad). Libraries were prepared for sequencing using the NEBNext^®^ UltraTM II DNA Library Prep Kit for Illumina^®^ (New England Biolabs, USA) and dual index adapters affixed with the NEBNext^®^ Multiplex Oligos for Illumina^®^. These libraries were then sequenced in a 250-bp paired-end run on the Illumina Novaseq6000 platform with the Novaseq6000_SP flow cell Reagent Kit (500 cycles).

#### Bioinformatics analysis

The metagenomic study was conducted utilising the Nephele 2.0 QIIME2 16S Amplicon platform (https://nephele.niaid.nih.gov/)^18^. The workflow employed was the Quantitative Insights into Microbial Ecology pipeline (QIIME2) 16 S Amplicon pipeline. Initially, raw paired-end FASTQ files generated from Illumina sequencing were uploaded to the Nephele platform. The QIIME2-based pipeline began with quality assessment and demultiplexing of reads, followed by trimming of adapter sequences and low-quality bases using DADA2 for denoising, error correction, and chimera removal. Key metrics such as base quality (Phred score > 30), GC content, base composition, adapter dimers, and ambiguous bases were closely examined. The ASV counts were rarefied to 52,543 reads, the lowest read count, before being used for downstream analyses. Subsequently, taxonomic classification was performed using a pre-trained Naïve Bayes classifier against the SILVA 138 reference database, targeting the V3-V4 region of the 16S rRNA gene. The outputs were downloaded for further downstream analyses and visualization. Alpha diversity (Shannon, Simpson, Chao1, and abundance-based coverage estimator, ACE) was calculated using PAST software 4.10^19^. Principal component analysis (PCoA) based on Bray-Curtis similarity of ASV abundance profiles was carried out to analyse the β-diversity measures with PAST 4.10. The statistical significance of clustering patterns from the PCoA was evaluated using PERMANOVA. One-way PERMANOVA was done to compare the β-diversity indices of microbiota between groups with significantly different survival rates, and ANOSIM was then applied to validate the consistency of group separations. The differentially abundant bacterial ASVs between differentially treated larval groups and the control group, and between groups having significantly different survival were determined through analysis of composition of microbiomes (ANCOM) pipeline, which accounts for the compositional nature of microbiome data and inherently controls for multiple comparisons^20^. Therefore, no additional FDR correction was applied. Functional predictions of the metagenomics dataset were made using PICRUSt2 for identifying KEGG orthologs, enzymes, and pathways^21–24^. Alpha diversity (Shannon, Simpson, Chao1, and abundance-based coverage estimator, ACE) of PICRUSt2 results was calculated using PAST software 4.10.

### Statistical analysis

The Shapiro-Wilk and Levene tests were conducted to assess the data normality and variance homogeneity for α-diversity measures. For normally distributed data, one-way ANOVA with Tukey’s HSD post hoc test was used to compare groups, while the Kruskal-Wallis test was applied to non-normal data. In detail, the Mann-Whitney U test compared the hatching rates of each treatment with the control. Two-way ANOVA with Tukey’s HSD post hoc test compared the effect of day and disinfectant on larval survival rates, protein content, catalase activity and GSH content through SPSS (version 16). The potential correlations between larval survival rates to catalase, protein content, GSH, taxonomic α-diversity measures and abundance of microbiota at each taxa level were estimated using the tidyverse package version 2.0.0^25^ in R software (version 4.5.0). Biomarkers with potentially significant correlations with survival rates were visualised using horizontal bar plots. The Mann-Whitney U test was applied to compare the α-diversity indices of the taxonomic metagenomics and functional metagenomics data of each treatment with the control and to compare the larval groups with significantly different survival rates; since each diversity metric involved a single summary statistic per sample, rather than individual functional features, no multiple comparison (FDR) correction was applied. Differentially abundant ASVs from the ANCOM pipeline were applied to compute the microbial dysbiosis (MD) index between the larval groups as per the suggested formula, where MD = log_10_ [(total abundance of ASVs increased compared to the other group)/ (total abundance of ASVs decreased compared to the other group)^26^. The abundance of gut microbes at each taxonomic level and the abundance ratio of different phyla were compared using the Kruskal-Wallis H test between these groups with significantly different survival. All these analyses were done through SPSS (version 16). Three-set and four-set Venn diagrams were made using the ASV feature table in Python 3.13.3 (Venn package v0.1.3; Matplotlib v3.10.3).

## Results

### Effects of egg disinfection on the hatching rates of *T. blochii*

**Fig. 2:**
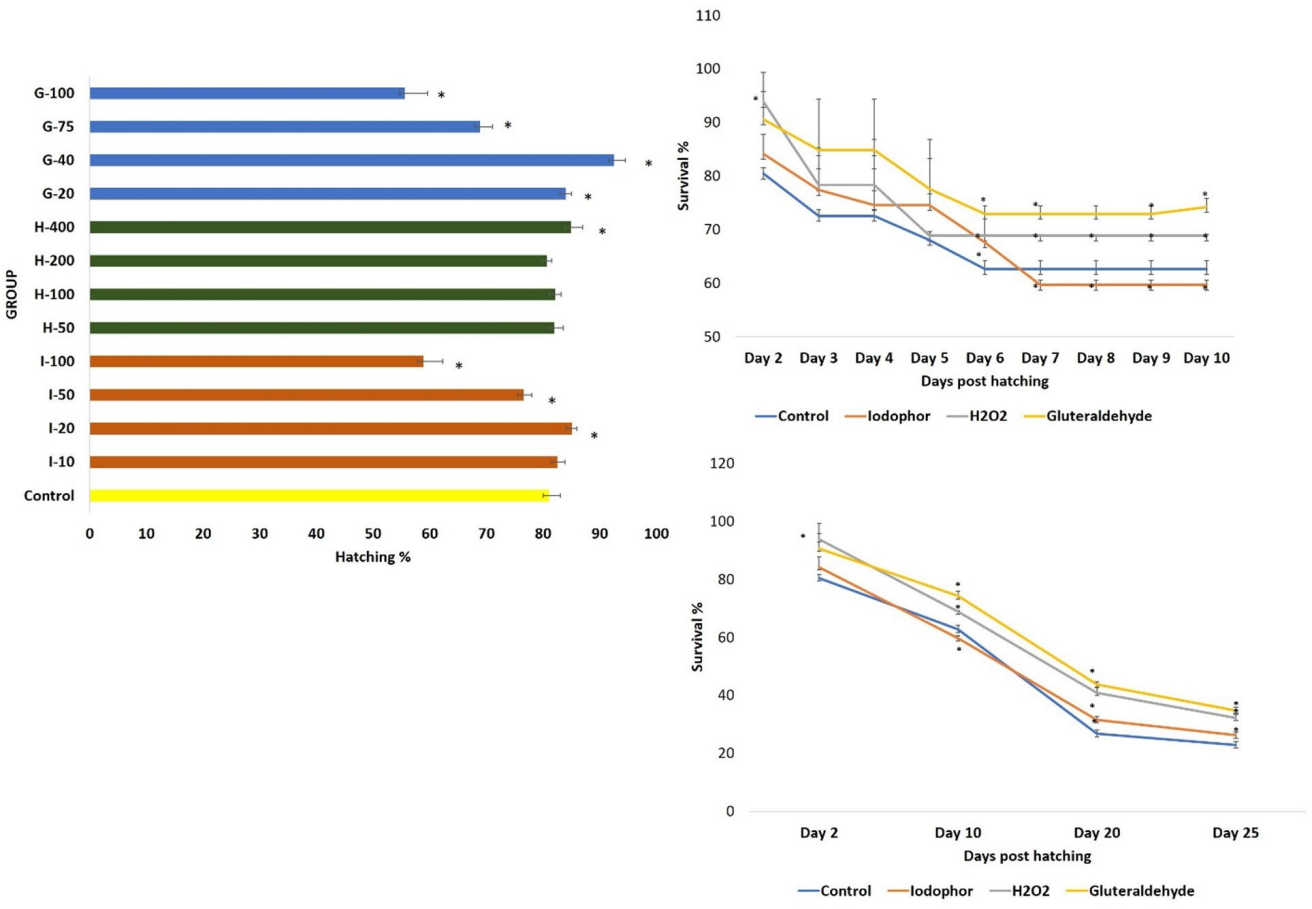
Effects of egg disinfection on the hatching and survival rates of *T. blochii.* (**A**) Effects on the hatching rates; (**B**) Effects on the survival rates up to 10 days post-hatching; (**C**) Effects on the survival rates up to 25 days post-hatching. In all figures, the values that are significantly different (*P* ≤ 0.05) from control group are indicated by *. Abbreviations used: G: Gluteraldehyde; I: Iodophor; H: Hydrogen peroxide. The numbers followed by the letters on the Y-axis of Fig. 2A indicates the concentrations of each compound in ppm

Among the tested concentrations of iodophor, higher doses (50 and 100 ppm) resulted in significantly lower (*P* ≤ 0.05) hatching rates compared to the control group (Fig. 2). In contrast, a notable improvement in hatchability was observed at 20 ppm. There was no significant difference in the hatchability between the 10-ppm group and the control group (Fig. 2). In the case of H_2_O_2_treatments, no significant differences (*P* ≥ 0.05) in the hatchability were found at 50, 100, and 200 ppm compared to the control. However, exposure to 400 ppm improved the hatching rate. Glutaraldehyde treatment at 20 and 40 ppm significantly enhanced hatching rates, whereas higher concentrations (75 and 100 ppm) caused a significant reduction (*P* ≤ 0.05). Among all disinfectant treatments, 40 ppm glutaraldehyde yielded the highest hatching rate (92.55 ± 1.4%), marking an approximate 15.38% increase over the control.

### Effects of egg disinfection on the survival rates of *T. blochii* larvae

**Fig. 3:**
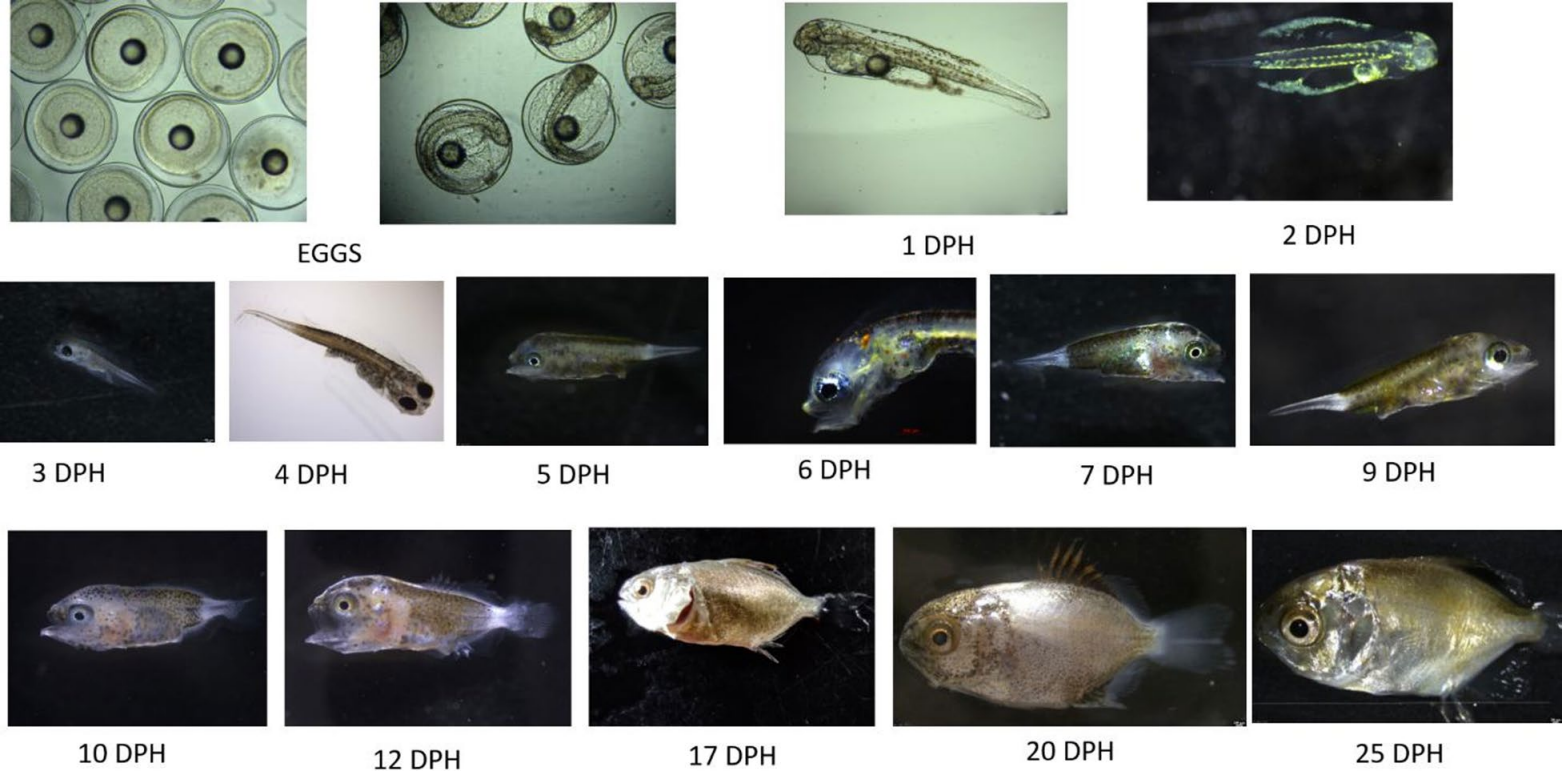
Representative photographs of *T. blochii* larvae exhibiting normal morphology. DPH: Days post-hatching.

The comprehensive experiments were done using the selected concentration-time combination of H_2_O_2_ (400 ppm for 10 min), iodophor (20 ppm for 10 min), and glutaraldehyde (40 ppm for 5 min). No larval deformities were detected in any of the groups (Fig. 3). Two-way ANOVA revealed that both days post hatching (DPH) and type of disinfectant had significant effects on survival rates (*P* ≤ 0.05), with no significant interaction between the two factors (*P* = 0.34). The highest mortality occurred within the first five days after hatching in the test and control groups. From the 6 DPH onwards, egg disinfection with glutaraldehyde, iodophor, and H_2_O_2_ significantly improved survival compared to the control group (Fig. 2). However, from the 7 to 10 DPH, the survival in iodophor-treated larvae was lower than in the control. On 20 DPH, egg disinfection with all tested chemicals significantly improved survival compared to the control group (Fig. 2). At 20 DPH, glutaraldehyde (43.74%) and H_2_O_2_ (40.87%) yielded comparable survival rates, both significantly higher than iodophor (31.56%) and the control (26.74%). By 25 DPH, glutaraldehyde treatment resulted in the highest survival (34.80 ± 1.1%), followed by H_2_O_2_, iodophor, and the control.

### Profiling the larval quality indices

**Fig. 4:**
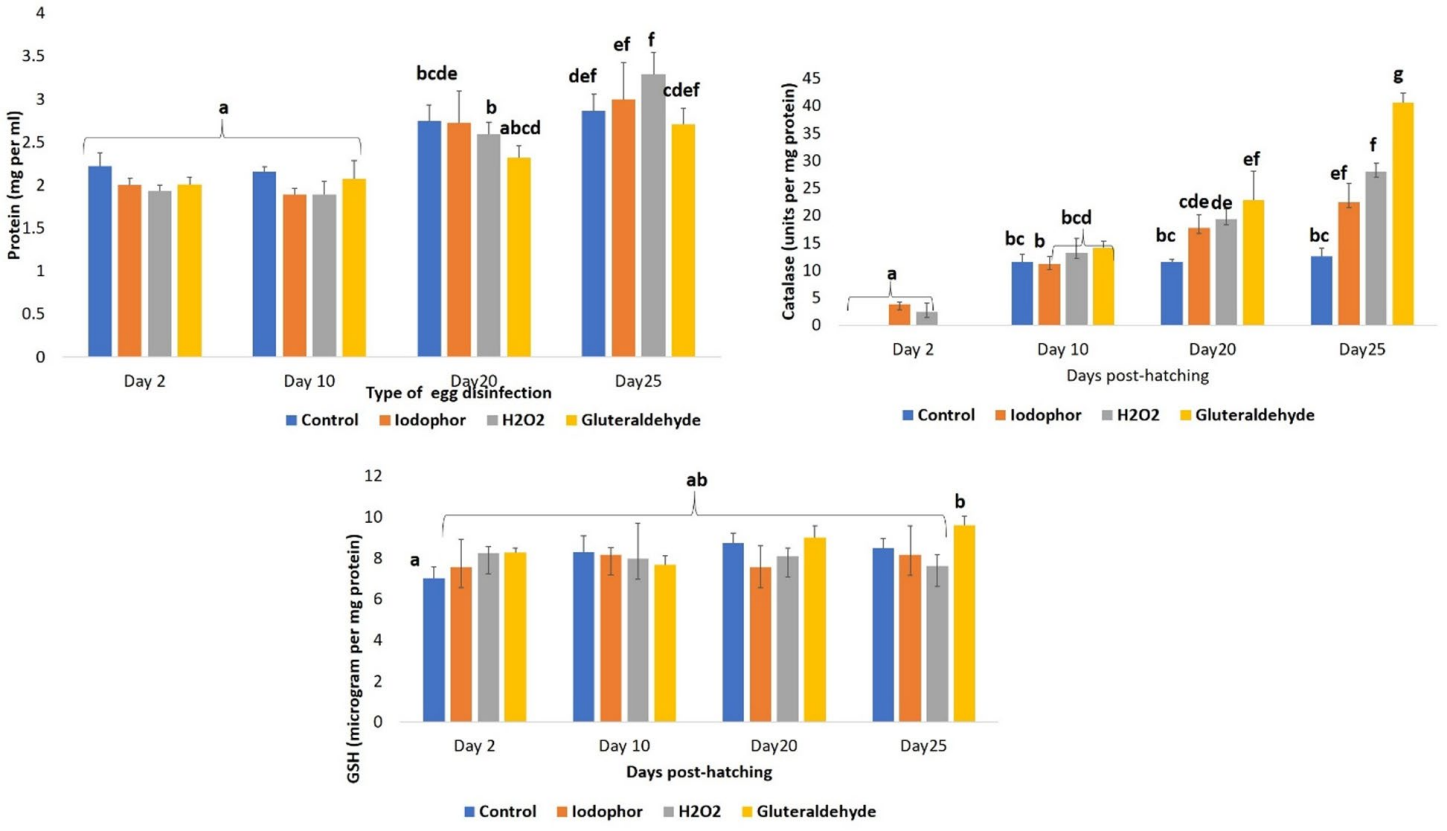
Effects of egg disinfection on the selected larval quality indices of *T. blochii*. (**A**) Protein content in larvae at different days post-hatching; (**B**) Catalase activity in larvae at different days post-hatching; Reduced glutathione content in larvae at different days post-hatching. In all figures, the values that are significantly different (*P* ≤ 0.05) from control group are indicated by *.

Two-way ANOVA revealed that only the day post-hatching had a significant effect (*P* ≤ 0.001) on larval protein content. There was no significant difference (*P* ≥ 0.05) in protein content between 2 and 10 DPH. But, the larvae at 20 and 25 DPH exhibited significantly higher protein levels compared to those at 2 and 10 DPH, with the highest protein content observed at 25 DPH (Fig. 4) in all groups.

**Fig. 5:**
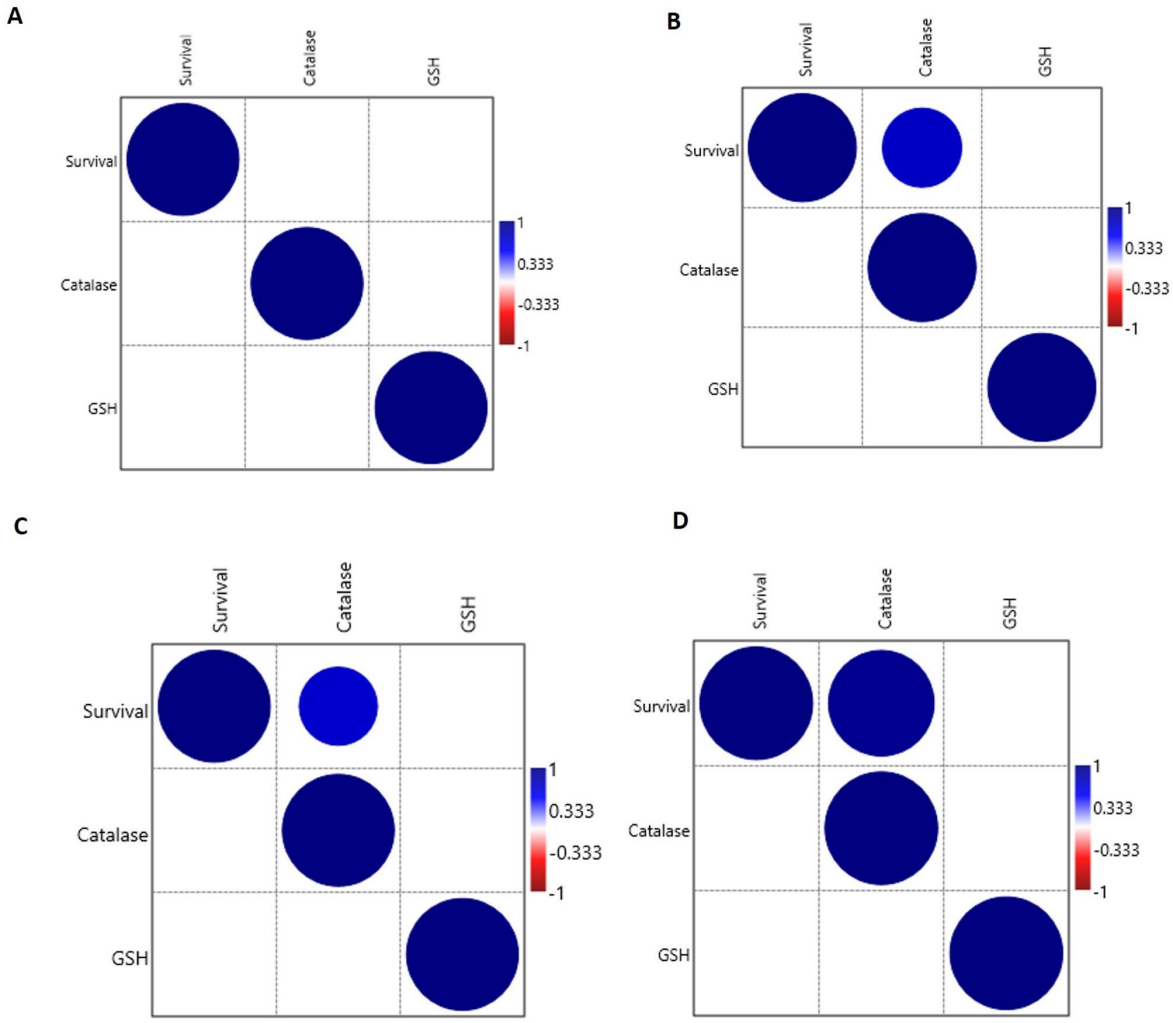
Correlation analysis of larval survival rates with different larval antioxidant profiles. (**A**) Kendall’s Tau correlations at 2 days post-hatching (DPH). (**B**) Kendall’s Tau correlations at 10 DPH; (**C**) Kendall’s Tau correlations at 20 DPH; (**D**) Kendall’s Tau correlations at 25 DPH. Significant positive correlations (*P* ≤ 0.05) were indicated by blue bubbles and the size of bubbles indicates the strength of correlation. Nonsignificant correlations were kept as blanks. GSH: Reduced glutathione content.

For catalase activity in larval homogenates, two-way ANOVA indicated that both DPH and disinfectant type had significant effects (*P* ≤ 0.001), with a significant interaction between the two factors (*P* ≤ 0.001). Catalase activity increased progressively with age, peaking at 25 DPH (Fig. 4). On 2 DPH, no catalase activity was detected in glutaraldehyde-treated and control larvae. However, on subsequent sampling days, glutaraldehyde-treated larvae exhibited the highest catalase activity, followed by those treated with H_2_O_2_, iodophor, and the control group. Regarding GSH content, neither DPH (*P* = 0.17) nor disinfectant type (*P* = 0.13) had significant effects (Fig. 4). Correlation analysis showed a significant correlation between catalase activity and survival rates on different DPH (10, 20, and 20 DPH), except for 2 DPH (Fig. 5).

### Metagenomics data set

All the samples qualified for the metagenomic library preparation and sequencing. The data sets were placed in the NCBI-SRA (Sequence Read Archive) database under Bioproject PRJNA765138 (Accession numbers: SRR34022291 to SRR34022302).

### Microbial diversity measures in whole larvae across different experimental groups

**Fig. 6:**
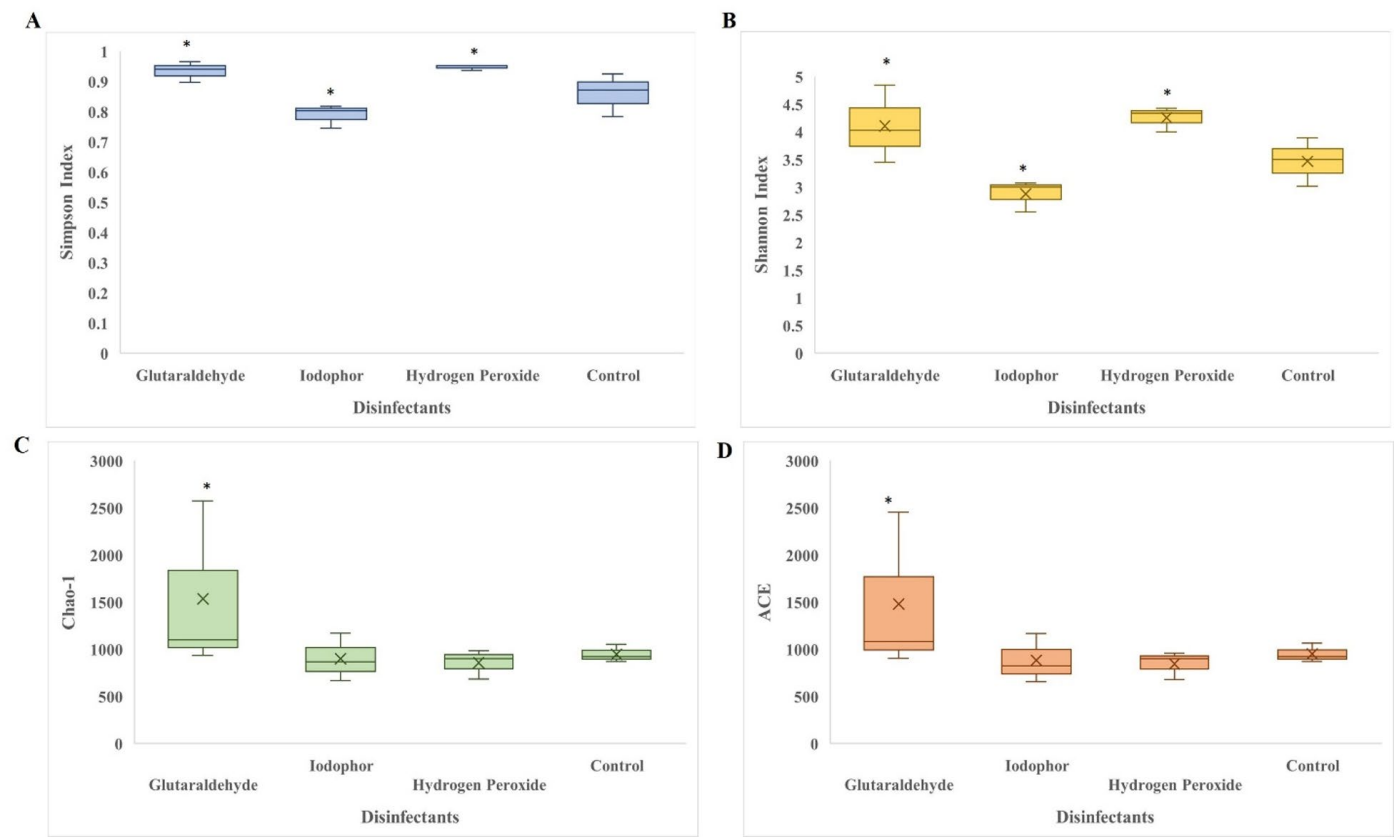
Effects of egg disinfection on the whole larval microbial taxonomic diversity measures at 10 days post-hatching. (**A**) Simpson index; (**B**) Shannon index; (**C**) Chao1 index; (**D**) Abundance-based Coverage Estimator. In all figures, the values that are highly significantly different (*P* ≤ 0.05) from control group are indicated by *.

Alpha-diversity measures of ASVs in 10 DPH larvae revealed significant differences among the treatment groups from the control (Fig. 6). Larvae treated with glutaraldehyde exhibited significantly higher diversity indices (*P* ≤ 0.05) compared to the control group.  H_2_O_2_-treated larvae showed significantly higher values for Shannon and Simpson indices *(P* ≤ 0.05). In contrast, iodophor-treated larvae displayed a significantly lower Shannon index and Simpson indices (*P* ≤ 0.05) than the controls. Notably, the larval group with higher survival rates as a whole (glutaraldehyde and H_2_O_2_-treatments) demonstrated significantly elevated α-diversity across all indices compared to the control group and the group with lower survival (iodophor-treated larvae).

**Fig. 7:**
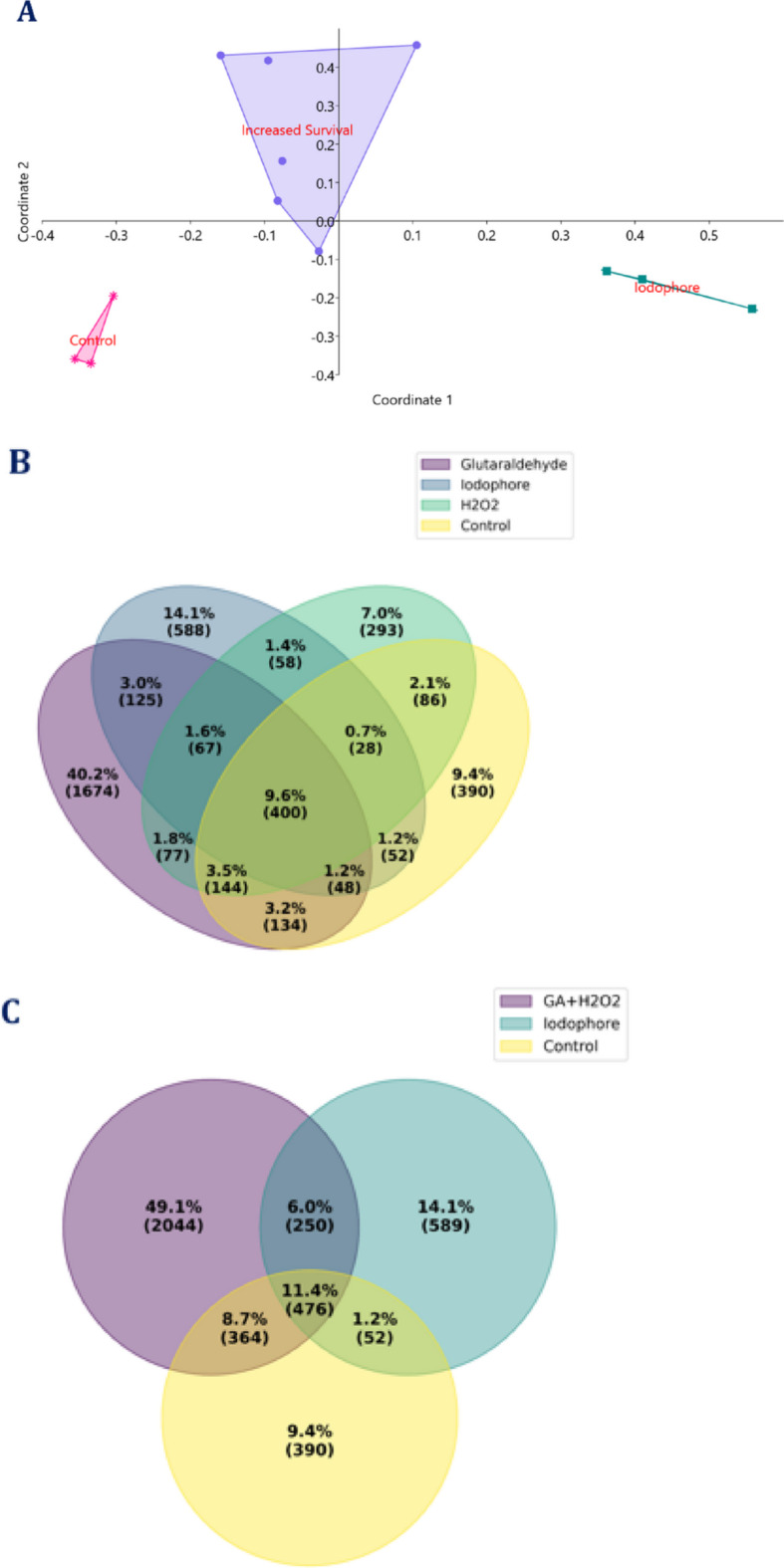
Comparison of microbial taxonomic profiles between different studied groups. (**A**) Principal Coordinate Analysis of whole larval microbiota profiles based on Bray-Curtis similarity distances; (**B**) Shared ASVs between different studied groups; (**C**) Shared ASVs between groups showing significantly different survival rates. In Fig. A, three significantly different clusters are shown as the larval microbiota profiles after hydrogen peroxide and glutaraldehyde treatment formed significantly similar (*P* ≥ 0.05) clusters. This cluster is named as the cluster with increased survival rates. In Fig. B and C, the details of ASVs shared between each group are included in Supplementary File 4. Abbreviations used: GA: Gluteraldehyde; H_2_O_2_: Hydrogen peroxide

PCoA based on the Bray-Curtis similarity index showed three well-differentiated clusters in the whole larval microbiota profiles (Fig. 7). Samples treated with iodophor were clustered on the far right of PCoA1, indicating that the treatment caused a shift in microbial composition, making it distinct from all other groups. Larvae treated with H_2_O_2_ and glutaraldehyde formed a statistically similar (*P* ≥ 0.05) cluster, but significant from the control and iodophor-treated larval samples. Control larval samples were located in the bottom left, distant from the iodophor and the other treated larval groups, forming a tight cluster. The PERMANOVA test confirmed the significant difference (*P* = 0.0004; *F* = 7.026) of the clustering pattern revealed through the PCoA. The pairwise PERMANOVA analysis showed significant differences in microbial community composition between the larval group with higher survival rates (glutaraldehyde and H_2_O_2_ treatments) and the control group (*P* = 0.012; *F* = 4.5). Further, there was a significant difference between the larval group with higher survival rates and iodophor-treated larvae (*P* = 0.01; *F* = 6.48). However, no significant difference between the control and iodophor-treated larvae was observed (*P* = 0.09). ANOSIM supported these findings (*R* = 0.81, *p* = 0.0003). The pairwise ANOSIM revealed significant differences in microbial community composition between the larval groups with higher survival rates (glutaraldehyde and H_2_O_2_ treatments) and control larvae (*P* = 0.011; *R* = 0.64). Further, there was a significant difference between the larval group with higher survival rates and iodophor-treated larvae (*P* = 0.01; *R* = 0.81). However, no significant difference between the control and iodophor-treated larvae was observed (*P* = 0.10).

### Taxonomic profiles of *T. blochii* larval microbiota at 10 DPH

Taxonomic assignment yielded 35 phyla, 75 classes, 197 orders, 374 families, and 749 genera across the whole microbiota profiles (Supplementary File 1). *Proteobacteria* (67.74%) was the most dominant phylum, followed by *Cyanobacteria* (23.78%) > *Bacteroidota* (3.58%) > *Firmicutes* (1.94%) > *Acidobacteriota* = *Myxococcota* (0.59%) > *Actinomycetota* (0.57%) > *Bdellovibrionota* (0.54%). The α-*Proteobacteria* (39.36%), *Cyanobacteria* (23.78%) > unassigned *Proteobacteria* (18.47%) > γ-*Proteobacteria* (9.33%), *Bacteroidia* (3.57%), and *Bacilli* (1.5%) occupied the six most dominant positions at the class level. The PICRUSt2 analysis showed 7728 KEGG genes, 431 KEGG pathways, and 2337 KEGG enzymes across the whole microbiota profiles of *T. blochii* (Supplementary File 2).

### Comparison of microbial taxonomic profiles between differentially treated groups

The ANCOM analysis revealed 118 ASVs differentially abundant between glutaraldehyde-treated larvae and control larvae, of which 54 were enriched and 64 depleted (Supplementary File 3). Differentially abundant markers occupied 28.1% and 23.7% of total abundance in the control and treated group, respectively. Similarly, 89 ASVs were differentially enriched, and 124 were depleted in iodophor-treated larvae compared to control larvae, occupying 38.09% and 75.23% abundance in the control and treated larvae, respectively. In H_2_O_2_-treated larvae, 104 ASVs were differentially enriched, and 66 were depleted compared to control larvae, occupying 33.02% and 33.7% abundance in the control and treated larvae, respectively. MD was calculated as 0.6, 1.1, and 0.7 for glutaraldehyde, iodophor, and H_2_O_2_-treated larvae, respectively. Approximately 9.6% of ASVs were shared across all the groups (Fig. 7; Supplementary File 4).

### Comparison of microbial taxonomic profiles between the groups showing significantly different survival rates

On the 10 DPH, the experimental groups formed three clusters based on survival rates. Larvae from the glutaraldehyde and H_2_O_2_-treated group showed significantly improved survival compared to the control group. The iodophor-treated group showed a significantly lower survival. Accordingly, a comparative analysis of these three clusters was done. Significant (*P* ≤ 0.05) alterations in certain gut microbial taxa at different taxonomic levels were noticed between the groups showing significant differences in survival (Table 1). Abundances of six phyla were significantly altered between the groups, of which abundances of three, namely *Bacteroidota*,* Myxococcota*, and *Bdellovibrionota*, were higher in the larval groups showing significantly increased survival, while significantly lower in groups with less survival. Further, the ratio between the abundance of *Proteobacteria* to *Firmicutes* (*P* ≤ 0.001), *Proteobacteria* to *Bacteroidota* (*P* = 0.03), *Firmicutes* to *Bacteroidota* (*P* = 0.02), and *Fusobacteriota + Firmicutes + Bacteroidota* to *Proteobacteria* were significantly (*P* = 0.01) altered between the groups. A lower *P* to *F* ratio, higher *P/B* ratio, and *F/B* ratio was observed in the iodophor group compared to the control. Even though a decreased *P* to *F* ratio and increased *Fusobacteriota + Firmicutes + Bacteroidota* compared to control were observed in the group with higher survival. At class, order, family and genus levels, abundances of 8, 29, 29, and 44 were significantly altered between the groups (Table 1).

**Table 1:** Major changes from the control group in relative abundance after egg disinfection.

Microbial group	Iodophor	Increased survival group
**At phyla level**		
*Proteobacteria*	↑	↑
*Cyanobacteria*	↓↓	↓↓
*Bacteroidota*	↓	↑↑
*Myxococcota*	↓	↑↑
*Bdellovibrionota*	↓	↑↑
*Actinomycetota*	↑↑	↑↑
*Proteobacteria* to *Firmicutes*	↓↓↓	↓↓↓
*Proteobacteria* to *Bacteroidota*	↑↑	NC
*Firmicutes* to *Bacteroidota*	↑↑↑↑	NC
*Fusobacteriota + Firmicutes + Bacteroidota* to *Proteobacteria*	NC	↑↑
**At class level**		
Unassigned *Proteobacteria*	↑↑↑	↑↑
*Cyanobacteria*	↓↓	↓↓
*Bacilli*	↑↑↑↑	↑↑↑↑
*Bacteroidia*	↓	↑↑
*Polyangia*	↓	↑
*Bdellovibrionia*	↓	↑↑
*Actinobacteria*	↑↑↑↑	↑↑
*Myxococcia*	↓	↑↑↑
**At order level**		
Unassigned *Proteobacteria*	↑↑↑	↑↑
*Cyanobacteriales*	↓↓	↓↓
*Rhizobiales*	↑↑↑	↑↑
*Caulobacterales*	↓↓	↑
*Rickettsiales*	↓↓↓↓	↓↓
*Sphingomonadales*	↓↓↓	↓
*Acetobacterales*	↑↑↑↑	↑↑
*Phormidesmiales*	↓↓↓↓	↓↓
*Bacillales*	↑↑↑↑	↑↑↑
*Acidobacteriales*	↑↑↑↑	↑↑↑
*Flavobacteriales*	↓	↑↑
*Cellvibrionales*	↓↓	↑
*Nannocystales*	↓	↑↑
*Parvibaculales*	↓↓↓	↓
*Cytophagales*	↓↓↓	↓↓
*Myxococcales*	↑	↑↑↑
*Rhodospirillales*	↓↓↓	↓
*Pseudomonadales*	↑↑↑	↑↑
*Micrococcales*	↑↑↑↑	↑↑
*Xanthomonadales*	↑↑↑↑	↑↑
*Corynebacteriales*	↑↑↑↑	↑↑
*SS1-B-07-19*	↓↓↓↓	↓↓↓↓
*Erysipelotrichales*	↑↑↑↑	↑↑↑↑
Unassigned *Bacteroidia*	↓↓↓↓	↑↑↑↑
*WPS-2*	↑	↑↑↑
*Micropepsales*	↑↑↑↑	↑↑↑↑
*Solirubrobacterales*	↑↑↑↑	↑↑↑↑
Subgroup 7	NC	↑↑↑↑
*Streptomycetales*	↑↑↑↑	↑↑↑↑
*WD260*	NC	↑↑↑↑
*GAL15*	NC	↑↑↑↑
**At family level**		
Unassigned *Proteobacteria*	↑↑↑	↑↑
*Cyanobacteriaceae*	↓↓	↓↓
*Rhizobiaceae*	↑↑	↑
*Hyphomonadaceae*	↓↓	↑
*AB1*	↓↓↓↓	↓↓↓
*Sphingomonadaceae*	↓↓↓	↓
*Acetobacteraceae*	↑↑↑↑	↑↑
*Phormidesmiaceae*	↓↓↓↓	↓↓
*Bacillaceae*	↑↑↑↑	↑↑↑
*Acidobacteriaceae* (Subgroup 1)	↑↑↑↑	↑↑↑
*Halieaceae*	↓↓↓	↑
*Nannocystaceae*	↓	↑↑
*Cyclobacteriaceae*	↓↓↓	↓↓
*Alteromonadaceae*	↓↓	↑↑
*Unassigned Myxococcales*	↑	↑↑↑
*Terasakiellaceae*	↓↓↓	↓
*Moraxellaceae*	↑↑↑↑	↑↑↑↑
*Weeksellaceae*	↑↑↑	↑↑
*Xanthomonadaceae*	↑↑↑↑	↑
*SS1-B-07-19*	↓↓↓↓	↓↓↓↓
*Intrasporangiaceae*	↑↑↑↑	↓
*Brevibacteriaceae*	↑↑↑↑	NC
*Microbulbiferaceae*	↓↓↓↓	↓↓↓↓
Unassigned *Micavibrionales*	↑↑↑↑	NC
*Dermabacteraceae*	↑↑	↑
*67 − 14*	↑↑↑↑	NC
*Rhodothermaceae*	↓↓↓↓	↓↓↓↓
*MB-A2-108*	↑↑↑↑	NC
**At genus level**		
Unassigned *Proteobacteria*	↑↑↑	↑↑
*Symphothece* PCC-7002	↓↓	↓↓
Unassigned *Rhizobiaceae*	↑↑↑	↑↑
Uncultured bacterium 1	↓↓↓	↑
*AB1*	↓↓↓↓	↓↓↓
*Erythrobacter*	↓↓↓	↓
*Acidisoma*	↑↑↑↑	↑↑
*Acrophormium PCC-7375*	↓↓↓↓	↓↓↓
*Bacillus*	↑↑↑↑	↑↑↑
*Edaphobacter*	↑↑↑↑	↑↑↑
Unassigned *Halieaceae*	↓↓↓	↓
Uncultured *Nannocystaceae*	↓	↑↑
Uncultured bacterium 2	↓↓↓	↓
*Labrenzia*	↑↑↑	↑↑
*Ekhidna*	↓↓↓	↓↓↓↓
*Unassigned Myxococcales*	↑	↑↑↑
*Cyanobacterium CLg1*	↓↓	↓↓↓↓
*Roseovarius*	↓↓	↓↓
Unassigned *Bradymonadales*	↑↑↑↑	↑↑
OM60 (NOR5) clade	↓↓	↑↑↑↑
*Roseivirga*	↑↑	↑↑↑↑
*Terasakiella*	↓↓↓↓	↓↓
*Enhydrobacter*	↑↑↑↑	↑↑↑↑
*Moheibacter*	↑↑↑↑	↑↑↑↑
*Corynebacterium*	↑↑↑↑	↑↑↑↑
*Neptuniibacter*	↓↓↓	↓↓↓↓
Unassigned *Bacillaceae*	↑↑↑↑	↑↑↑↑
*SS1-B-07-19*	↓↓↓↓	↓↓↓↓
*Nitrincolaceae*	↑↑↑↑	NC
JG30-KF-CM45	↑↑↑	↓↓↓
*Coraliomargarita*	↓↓↓↓	↓↓↓↓
*Pseudogracilibacillus*	↑↑↑↑	↑↑↑↑
Uncultured bacterium 3	↑↑↑↑	NC
Uncultured bacterium 4	↑↑↑↑	NC
*Brevibacterium*	↑↑↑↑	↑↑↑↑
Unassigned *Sphingobacteriaceae*	↑↑↑↑	↑↑↑↑
67 − 14	↑↑↑↑	NC
*Bermanella*	↓↓↓↓	↓↓
*Keratinibaculum*	↑↑↑↑	NC
*Enteractinococcus*	↑↑↑↑	NC
*MB-A2-108*	↑↑↑↑	NC
*Cryptosporangium*	↓↓↓↓	↓↓↓↓
*Porticoccus*	↓↓↓↓	↓↓↓↓

Only those taxa showing significant changes in relative abundance compared to the control group are shown. Upward arrows indicate the increase in the relative abundance compared to control group, where one, two, three, and arrows show the increase ≤ 2, 2–5, 5–10, and > 10 times, respectively higher than the control group. Downward arrows indicate the decrease in the relative abundance from the control group, where one, two, three, and arrows show the increase ≤ 2, 2–5, 5–10, and > 10 times, respectively lower than the control group. NC, no significant change (*P* ≤ 0.05) from the control.

ANCOM analysis revealed 80 differentiating microbial markers between the groups, showing significant differences in survival rates, of which the abundance of 31 was significantly higher in the group with higher survival (Supplementary File 3). Particularly, the abundances of *Photobacterium*,* Vibrio*,* Erythrobacter*,* Halieaceae*, and certain *Rhodobacteraceae* were enriched. The abundances of certain other *Rhodobacteraceae* and uncultured *Marinobacterium* were depleted. The MD index calculated for the group with higher survival was 0.42 compared to the control group. The differentiating markers formed an abundance of 21% in the control and 9% in the increased survival group, respectively. It was found that the abundances of > 80% larval microbes remained unchanged between the two groups.

ANCOM analysis revealed 112 differentiating microbial markers between the groups, showing significant differences in survival rates from iodophor-treated larvae. Abundances of 64 were significantly higher in the group with higher survival (Supplementary File 3). Particularly, the abundances of *Rhodobacteraceae*, uncultured *Sphingobacteriales*,* Porphyrobacter meromictius*,* Erythrobacter*, and *Halieaceae* were enriched. The abundances of *Rhizobiaceae*, various *Rhodobacteraceae*, and *Shimia*,* Candidatus endobugula*, and *Staphylococcus carnosus* were depleted. Venn diagram analysis showed that 476 (11.4%) ASVs were shared across the three categories. Additionally, another 364 (8.7%) were shared between groups with higher survival and control (Fig. 7; Supplementary File 4).

### Correlation analysis between the survival and metagenomic data

**Fig. 8:**
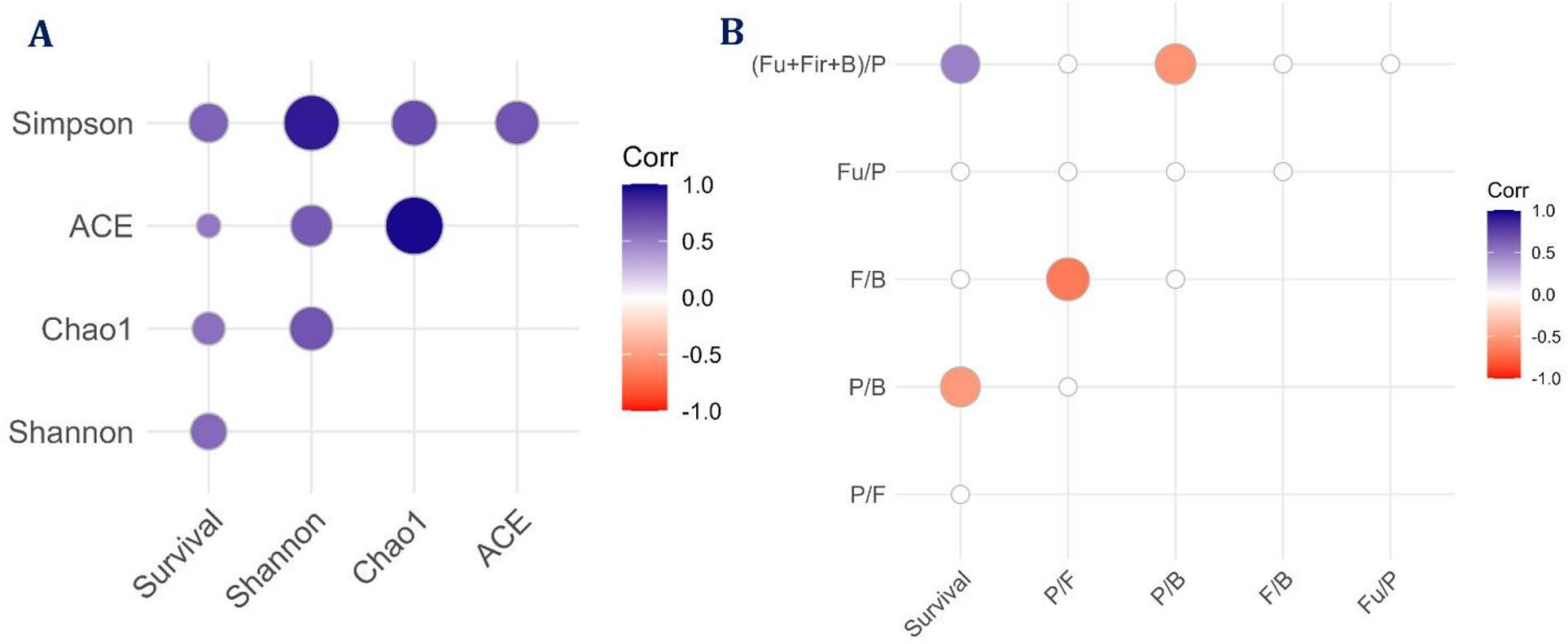
Kendall’s Tau correlation analysis between the whole larval microbiota taxonomic profiles and survival rates at 10 days post-hatching. (**A**) Kendall’s Tau correlations between survival and taxonomic diversity features of whole larval microbiota at 10 DPH; (**B**) Kendall’s Tau correlations between survival and the ratio between the abundance data of different phyla of whole larval microbiota at 10 DPH. Significant positive correlations (*P* ≤ 0.05) are indicated by blue bubbles, and negative correlations are indicated by red coloured bubbles. The size of bubbles indicates the strength of correlation. Nonsignificant correlations were kept as blanks. Fu: *Fusobacteriota*; Fir: *Firmicutes;* B: *Bacteroidota*; P: *Proteobacteria*; Corr: Kendall’s Tau correlation coefficient.

**Fig. 9:**
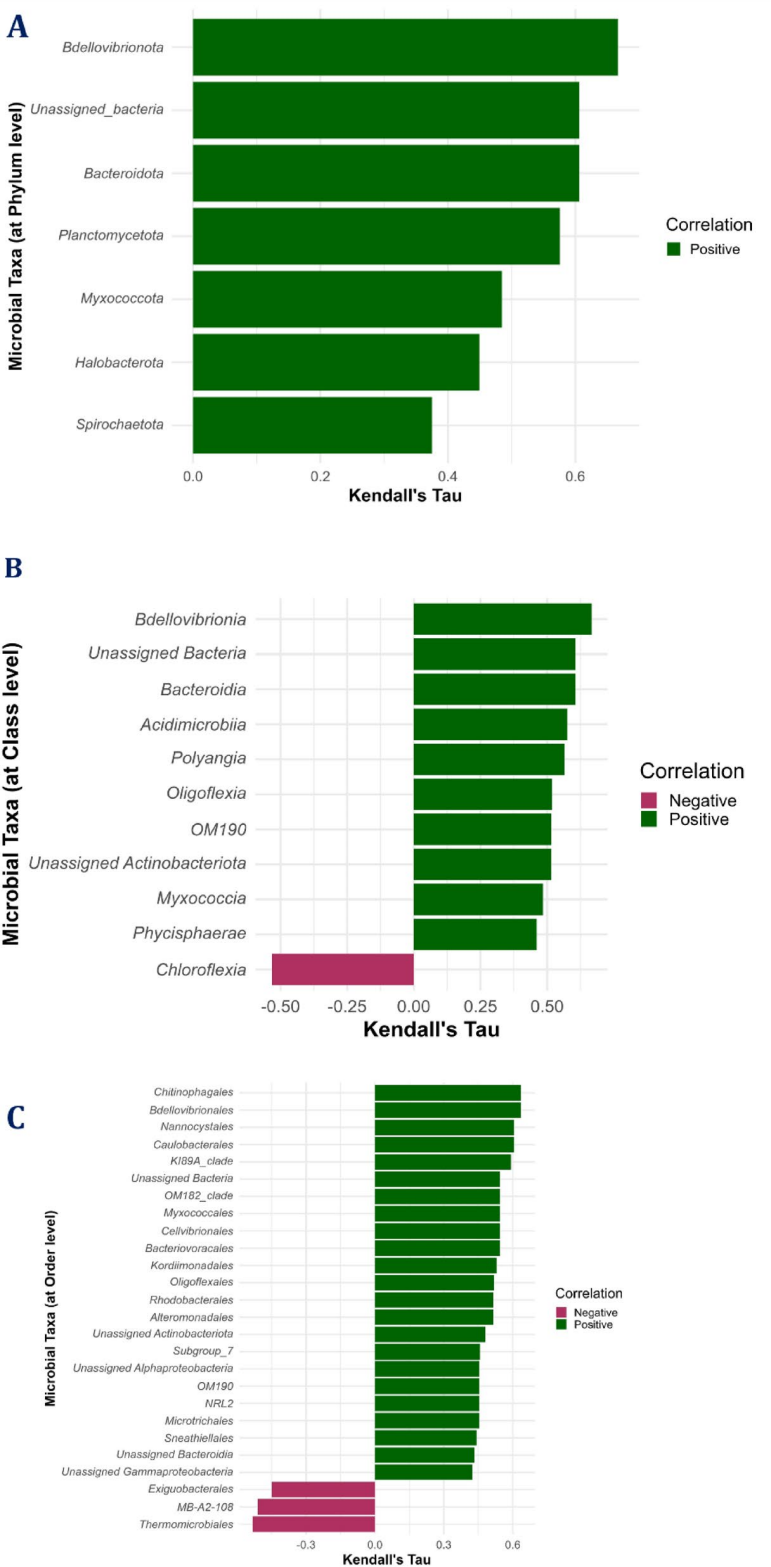

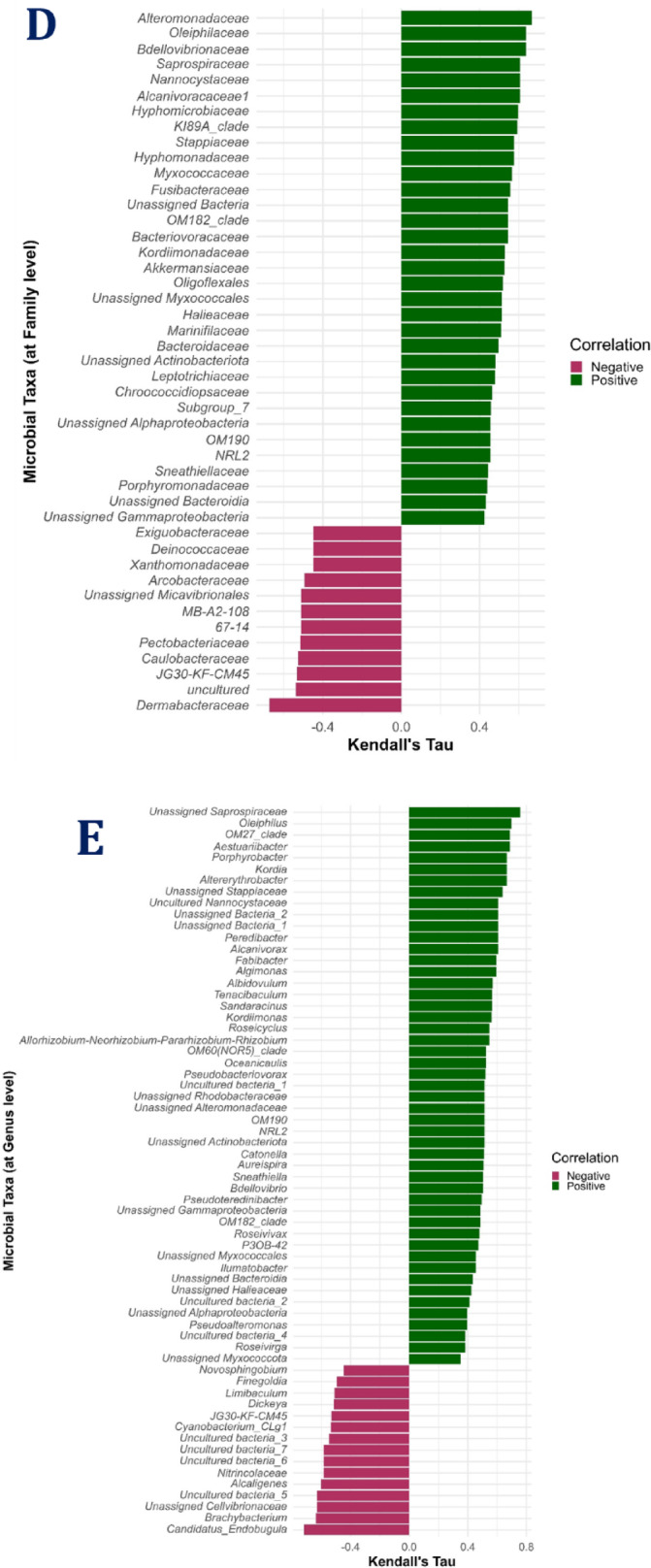
Kendall’s Tau correlation analysis between the ASV features and survival rates at 10th days post-hatching. (**A**) At phylum level; (**B**) At class level; (**C**) At order level; (**D**) At family level; (**E**) At genus level. Significant correlations (*P* ≤ 0.05) were only shown.

Correlation analysis showed positive significant correlations of survival rates with all taxonomic α-diversity measures with maximum correlation with the Simpson index followed by Chao1 (Fig. 8). At the phylum level, there was a significant positive correlation of survival with the abundances of *Bacteroidota*,* Myxococcota*,* Bdellovibrionota*,* Halobacterota*,* Planctomycetota*, and one unassigned bacterium (Fig. 9). The most abundant phylum *Proteobacteria* showed a positive significant correlation with *Firmicutes*,* Acidobacteriota*, and *Actinomycetota* and a negative correlation with *Cyanobacteria* (Supplementary Fig. 5). Notably, the *Proteobacteria* to *Bacteroidota* ratio showed a significant negative correlation with larval survival. Further, (*Fusobacteriota* + *Firmicutes* + *Bacteroidota*): *Proteobacteria* ratio showed a significant positive correlation with larval survival (Fig. 8). The various taxa showing significant correlations with survival rates at different taxonomic levels (class, order, family, and genus) are represented in Fig. 9.

### Comparison of functional metagenomic profiles of the whole larvae between different experimental groups

Alpha-diversity measures of functional metagenomic profiles of  10 DPH larvae revealed significant differences among the treatment groups (Table 2). Larvae treated with glutaraldehyde and H_2_O_2_ exhibited significantly higher Simpson and Shannon indices (*P* ≤ 0.05) of KEGG genes and pathways compared to the control group. In contrast, iodophor-treated larvae displayed significantly similar indices of KEGG genes, pathways, and enzymes to the controls. Notably, the larval group with higher survival rates (glutaraldehyde and H_2_O_2_  treatments) demonstrated significantly elevated Simpson and Shannon indices of KEGG genes, pathways, and enzymes compared to the control group and the group with lower survival (iodophor-treated larvae).

**Table 2:** α-diversity measures of functional taxonomic profiles.

KEGG Genes	Glutaraldehyde	Iodophor	H_2_O_2_	Increased survival	Control
Shannon’s H diversity index	7.83^ab^ ± 0.07	7.77^a^ ± 0.04	7.90^b^ ± 0.02	7.87^ab^ ± 0.04	7.68^a^ ± 0.03
Simpson’s index	1.00^bc^ ± 0.00	1.00^a^ ± 0.00	1.00^c^ ± 0.00	1.00^bc^ ± 0.00	1.00^a^ ± 0.00
Chao1	7012.33^a^ ± 321.90	6736.33^a^ ± 303.79	6381.33^a^ ± 329.12	6696.83^a^ ± 229.27	6466.67^a^ ± 71.81
ACE	6972.33^a^ ± 325.72	6725.00^a^ ± 322.69	6363.67^a^ ± 314.44	6668.00^a^ ± 230.83	6474.00^a^ ± 82.71
**KEGG enzymes**					
Shannon’s H diversity index	6.79^a^ ± 0.04	6.76^a^ ± 0.02	6.81^a^ ± 0.01	6.80^a^ ± 0.02	6.68^a^ ± 0.02
Simpson’s index	1.00^a^ ± 0.00	1.00^a^ ± 0.00	1.00^a^ ± 0.00	1.00^a^ ± 0.00	1.00^a^ ± 0.00
Chao1	2131.33^a^ ± 78.44	2078.33^a^ ± 85.80	2012.67^a^ ± 63.14	2072.00^a^ ± 48.77	2038.33^a^ ± 10.60
ACE	2133.00^a^ ± 70.49	2076.00^a^ ± 89.08	2007.67^a^ ± 67.52	2070.33^a^ ± 43.51	2046.33^a^ ± 7.37
**KEGG pathways**					
Shannon’s H diversity index	5.46^b^ ± 0.05	5.44^a^ ± 0.06	5.49^b^ ± 0.01	5.48^b^ ± 0.02	5.38^a^ ± 0.01
Simpson’s index	1.00^bc^ ± 0.00	0.99^a^ ± 0.00	1.00^c^ ± 0.00	1.00^bc^ ± 0.00	0.99^a^ ± 0.00
Chao1	403.00^a^ ± 14.18	397.00^a^ ± 11.36	385.67^a^ ± 8.08	394.33^a^ ± 7.01	389.67^a^ ± 3.79
ACE	403.00^a^ ± 14.18	397.00^a^ ± 11.36	385.67^a^ ± 8.08	394.33^a^ ± 7.01	389.67^a^ ± 3.79

Average values with different lowercase letters as superscripts represent levels that are significantly different at *P* ≤ 0.05.

## Discussion

Optimising egg disinfection protocols is vital for improving larval survival and resilience, thereby supporting sustainable marine hatchery production. In this study, we assessed the efficacy of three common disinfectants for egg pretreatment namely, H₂O₂, glutaraldehyde, and iodophor in *T. blochii*, a high-value tropical mariculture species.

### Optimised disinfectant concentrations enhanced hatchability in *T. blochii* eggs

Hatchability is a sensitive indicator of the suitability of egg disinfectants in finfish hatcheries^27^. Accordingly, an initial experiment was conducted to identify optimal concentrations of disinfectants by assessing their impact on the hatching success. Iodophor was tested at 10, 20, 50, and 100 ppm; H₂O₂ at 50, 100, 200, and 400 ppm; and glutaraldehyde at 20, 40, 75, and 100 ppm. Of these, immersion in 20 ppm iodophor for 10 min, 400 ppm H₂O₂ for 10 min, and 40 ppm glutaraldehyde for 5 min significantly improved hatching success, with glutaraldehyde yielding the highest hatchability (90.88 ± 2%), representing a ~ 11% increase from the control. Similar superiority of glutaraldehyde was reported earlier for marine fish eggs^28^. Importantly, the absence of pH adjustment during iodophor treatment caused 100% embryo mortality, consistent with the pH-dependent toxicity of iodine to fish embryos^29^, emphasising the critical importance of monitoring water chemistry during disinfection. At low pH, free iodine and hypoiodous acid dominate; both are highly membrane-permeable and cytotoxic^30]– [31^. Further, as iodophors are formulated with a pH of 2 to 4 to maximize antimicrobial activity^32^, this increases the proportion of toxic free iodine and also causes a sudden drop in water pH. The combined effects of low pH and elevated free iodine likely explain the observed lethality.

Higher concentrations of iodophor and glutaraldehyde reduced hatchability even after pH adjustment, confirming their dose-dependent toxicity, as documented for different marine and freshwater species^6,28^. The World Organisation for Animal Health^33^ recommends 100 ppm of active iodine for 10 min to improve the hatchability of salmonid eggs. While temperate fish eggs tolerate higher doses (100 to 500 ppm iodophor for 15 to 30 min; 200 to 600 ppm glutaraldehyde for 4 to 10 min; 400 to 566 ppm H₂O₂ for 15 to 60 min)^2,3,33–35^, *T. blochii* eggs appear more sensitive except for H₂O₂. The present results and previous studies on temperate fish^36^ indicated that 100 to 400 ppm of H_2_O_2_ can be used for egg disinfection of tropical and temperate fish species, with minor variation between species. However, there was an increased toxicity for iodophor and glutaraldehyde at higher water temperatures and salinities, similar to previous studies^37^. Prior studies in tropical ornamental fish species and grouper eggs reported toxicity at 15 to 100 ppm iodine^27,38^, aligning with our results. Elevated temperature and salinity in tropical waters may exacerbate toxicity by enhancing uptake due to increased metabolic activity^39^, reactive oxygen species generation, and disinfectant instability, like glutaraldehyde, leading to the formation of more toxic intermediates^40^. However, a detailed comparison of the data across studies is complicated by differences in the developmental stage at which disinfection is applied, as oocyte permeability and activation status vary widely^3^. Overall, our findings highlight the need for species- and environment-specific optimization of egg disinfection protocols in tropical marine aquaculture.

### Egg disinfection improved the survival rates of *T. blochii* larvae

Studies on the effects of egg disinfection on the later life stages of marine fish are limited, with one report in *Hippoglossus hippoglossus* showing improved larval survival after glutaric dialdehyde treatment (400 ppm, 10 min)^41^. Similar benefits have been documented in freshwater species using active iodine (150 ppm, 30 min) in Atlantic salmon^42^ and H_2_O_2_ (400 ppm, 60 min) in *Misgurnus anguillicaudatus*^2^. In the present study, larval survival of *T. blochii* was significantly improved in glutaraldehyde- and H_2_O_2_-treated groups from 6 DPH onwards, whereas iodophor treatment reduced survival. Interestingly, on 20 DPH, all disinfectant treatments outperformed the control, with glutaraldehyde (43.74%) and H_2_O_2_ (40.87%) showing the highest survival, followed by iodophor (31.56%) and the control (26.74%). This pattern persisted through 25 DPH, with glutaraldehyde yielding the best outcome (34.80%). These results are consistent with earlier studies demonstrating the higher efficacy and lower toxicity of glutaraldehyde and hydrogen peroxide when applied at optimised concentrations and exposure durations^2,6,28^. Two-way ANOVA confirmed significant effects of both disinfectant type and observation day on survival, with no interaction, indicating that the effects of each disinfectant on survival remained consistent across time points. Overall, these findings demonstrated the effectiveness of egg disinfection in improving larval survival in *T. blochii* and identified glutaraldehyde and H_2_O_2_ as the most effective disinfectants under the tested conditions.

### Egg disinfection modulated the antioxidant status of *T. blochii* larvae

Nutritional cues and non-nutritional environmental stressors experienced during early developmental stages can influence postnatal growth, metabolism, and overall health in fish. While egg disinfection has been studied mainly for its effects on hatching and, to a lesser extent on survival in several fish species, little is known about its impact on larval physiology and microbiota. To date, only one study in *Misgurnus anguillicaudatus* examined antioxidant responses following egg disinfection, although no microbiota analyses were conducted^2^. In this study, we assessed catalase activity and GSH levels in *T. blochii* larvae post-disinfection, with protein content used for normalisation since the water content in fish larvae varies with ontogenetic stage^16^. Protein levels increased with larval development, reflecting enhanced anabolic activity and tissue development. Catalase activity varied with both larval stage and disinfectant type, with a significant interaction, indicating that the antioxidant response to disinfection varied depending on larval stages. The observed increase in catalase activity with oncogenic progression probably represents a maturing antioxidant defence system to meet rising metabolic demands and oxidative challenges during development, as reported earlier^16^. On 2 DPH, the iodophor-treated group revealed the highest catalase activity, probably reflecting an acute stress response. However, as larvae matured, those treated with glutaraldehyde constantly showed the highest catalase, consistent with findings in *Cyprinus carpio* and *Micropterus salmoides* after H₂O₂ egg pretreatment^8]– [9^. Jia et al.^10^ reported that short, moderate H₂O₂ exposure enhanced antioxidant enzymes in *C. carpio*, while prolonged, high doses suppressed them. Similarly, Wang et al.^2^ showed that egg pretreatment programmed antioxidant responses in *M. anguillicaudatus*. Catalase activity also correlated positively with larval survival, supporting its role as a biomarker of robustness. In contrast, GSH remained stable across DPH and treatments, aligning with earlier observations on relatively stable GSH levels across different larval stages in *T. blochii*^16^. The results suggest that GSH homeostasis is tightly regulated and may not be a sensitive indicator of oxidative stress in early development. The improved hatching rates, antioxidant status, and survival likely reflect reduced microbial loads on disinfected eggs. H₂O₂ oxidises microbes and enhances oxygen diffusion, iodophor releases free iodine to denature microbial proteins, and glutaraldehyde crosslinks microbial proteins, thereby reducing microbial loads^43–45^. The reduced microbial pressure on the egg surfaces may improve oxygen diffusion, which likely explains the observed improvements in hatching and catalase activity. Overall, the results of egg disinfection modulated catalase activity with beneficial effects on larval survival. It should be noted that the antioxidant response was evaluated using catalase activity and GSH levels only in the present study. While these parameters provide meaningful insights into cellular redox balance, the absence of additional markers such as superoxide dismutase activity, glutathione peroxidase activity, or lipid peroxidation indices limits broader physiological interpretations. Accordingly, the conclusions are confined to the antioxidant responses represented by catalase and GSH, and further investigations incorporating a wider panel of oxidative stress biomarkers are warranted in future studies to have a more comprehensive evaluation of the oxidative status.

### Egg disinfection modulated the taxonomic composition and functional profiles of larval microbiota

Egg pretreatments significantly influenced microbial diversity, community structure, and functional profiles of *T. blochii* larvae at 10 DPH. α-diversity was significantly higher in glutaraldehyde and H₂O₂-treated larvae, but lower in iodophor-treated groups compared to controls. The increased diversity likely reflects suppression of fast-growing pathogens on eggs, allowing balanced colonisation by beneficial taxa, a feature linked to improved survival in K-selected larviculture systems^46^. Nevertheless, the instantaneous influences of disinfection on egg microbiota and immediately post-hatching larvae were not assessed in this study, warranting further investigation to validate this hypothesis. Functional predictions (PICRUSt2) also revealed a greater diversity measure of KEGG genes, pathways, and enzymes in glutaraldehyde and H₂O₂-treated larvae, suggesting a more functionally diverse microbiota in these groups, aligning with their higher survival rates. Although these predictions are indirect and may be less reliable in marine microbiomes, where horizontal gene transfer and strain-level variability are common, the results suggest that egg disinfection can shape larval microbiota function, warranting future validation studies with shotgun metagenomics, metabolomics, or targeted enzyme assays.

β-diversity also confirmed that disinfectant treatments altered the microbial community structure, with iodophor-treated larvae forming a statistically distinct cluster, while glutaraldehyde and H_2_O_2_ clustered together. Comparative analysis of microbial taxonomic profiles also revealed a broad-scale community restructuring associated with survival outcomes. Interestingly, iodophor-treated larvae exhibited higher *Proteobacteria* to *Bacteroidota* (P/B) and *Firmicutes* to *Bacteroidota* ratios, which are metagenomics stress indicators in fish larvae^23,47,48^, along with a lower *Fusobacteriota* + *Firmicutes* + *Bacteroidota* to *Proteobacteria* ratio, another health indicator in fish^49^, supporting a shift toward a potentially unfavourable microbial environment in the iodophor-treated group. Conversely, the higher survival group contained an increased abundance of *Hyphomonadaceae*, *Halieaceae*, *Nannocystaceae*, and *Alteromonadaceae*. Of which *Alteromonas macleodii* is a recognised probiotic bacterium which improved the survival of cultured marine animals^50^. Additionally, the abundance of *JG30-KF-CM45* was significantly increased in larval group with increased survival. Earlier studies have shown the negative correlation of the abundance of this genus with the C-N ratio^51^ and the negative correlation of C-N ratios with fish health^52^, supporting our results. Despite all these shifts, > 80% of taxa were shared among groups, indicating the presence of a core microbiota that provides foundational functions irrespective of survival status. The α and β diversity measures, analysis of shared microbes through the Venn diagram and ANCOM, and MD index reinforced the hypothesis that iodophor treatment caused the maximum disruption of the beneficial microbial community, which could be linked to reduced larval survival. Notably, the iodophor treatment improved hatching (85.05% compared to 81.03% in control) but reduced larval survival, likely due to residual iodine toxicity affecting animals or beneficial microbes, as suggested in previous studies^53–55^. Such dysbiosis may explain the poorer outcomes in this group. However, there is no data for direct comparison due to the lack of studies on the residual toxicity of egg disinfection on the larvae or larval microbial community. Overall, glutaraldehyde and H₂O₂ treatments promoted a more diverse and functionally versatile microbiota associated with better survival at 10 DPH, likely by reducing opportunistic ‘r-strategist’ bacteria on the egg surface and favoring beneficial ‘K-strategist’ colonizers, which is a proposed method for managing microbial communities of larviculture^56^. These findings underscore the importance of considering microbiota responses when evaluating egg disinfection protocols in marine aquaculture.

### Correlation analyses showed microbiota profiles associated with larval quality

The correlation analyses uncovered significant associations of larval survival with several specific microbiome features, suggesting their potential as larval health indices. Most importantly, survival rates showed a strong positive association with α-diversity, consistent with the reports of decreased α-diversity of microbial taxonomics in stress^26^. Among the different diversity metrics, the Simpson index exhibited the strongest correlation, suggesting that microbial evenness has a critical role in larval health, an observation similar to plant-bacterial relations^57^. The increased complementarity between species due to higher evenness enhances resource utilization and ecosystem stability^2^, which might be the possible explanation for the observation.

At the phylum level, *Bacteroidota*, *Myxococcota*, and *Bdellovibrionota* correlated positively with survival, consistent with their roles in nutrient cycling and pathogen control in aquaculture^58]– [59^. Particularly, members of *Myxococcota* and *Bdellovibrionota* are well-known for their predatory lifestyle and have shown great potential as biocontrol agents in relevant animal models^60^. Families, including *Halieaceae*, *Rhodobacteraceae*, and *Saprospiraceae*, correlated positively with survival, consistent with their reported roles in the efficiency of biofloc systems^61^. Notably, *Rhodobacteraceae* have been recognized as effective probiotics in aquaculture to promote beneficial interactions and suppress pathogens^61]– [62^, warranting further investigations to identify the precise members of this family for probiotic development in larviculture. Several unassigned and candidate taxa (OM190, NRL2, OM182, etc.) also displayed significant correlations with survival rates, pointing to the relevance of yet-uncultured or understudied microbial members in shaping fish larvae-microbiota interactions. Briefly, these associations suggest possible potential targets for probiotic development in *T. blochii* larviculture. In contrast, survival was negatively correlated with the *Proteobacteria*-*Bacteroidota* ratio, while the (*Fusobacteriota* + *Firmicutes* + *Bacteroidota*)/*Proteobacteria* ratio correlated positively, highlighting the importance of balanced microbial communities rather than dominance by any one phylum^63^. Previous studies have linked these ratios to stress in fish^49,64,65^, aligning with our results. Overall, higher diversity, enrichment of specific beneficial taxa, and ratios between the abundances of certain phyla like *Proteobacteria* to *Bacteroidota* (negative correlation), ratio between (*Fusobacteriota* + *Firmicutes* + *Bacteroidota*): *Proteobacteria* (positive correlation) were linked with improved larval survival. Simultaneously, the observed relationships are correlative only, and future experimental validation involving controlled manipulation of the identified microbial communities or functional validation is needed to establish causality.

### Limitations

In this study, we used three biological replicates as pooled larval samples randomly collected from each tank. This sampling design ensured independence at the tank level and incorporated randomization during sampling, and was consistent with several published fish larval microbiome studies^4,48,66–69^, where resource limitations and logistical challenges often constrain large-scale replication. Nevertheless, a greater replication would improve statistical power and should be prioritised in future work to confirm and validate these results. Another limitation of the present study is that larval malformation was not systematically assessed, despite reports of disinfectant-induced developmental deformities in fish^70,71^. Future investigations should evaluate sublethal endpoints, including skeletal and morphological abnormalities, to provide a more comprehensive understanding of larval quality after egg disinfection. Investigations incorporating a wider panel of oxidative stress biomarkers are also warranted in future to have a more comprehensive evaluation of the oxidative status. Finally, we did not profile the microbiome of rearing water or tank surfaces, which may influence larval recolonisation. Including environmental microbiome data in future studies will help clarify how disinfectants shape host-microbe interactions.

## Conclusion

The present study demonstrated that pre-treatment of *T. blochii* eggs with selected disinfectants significantly modulated larval microbiota, affecting survival and antioxidant responses. Based on our results, we recommend single dip treatments at the optic vesicle stage (8 h post-spawning) using H_2_O_2_ (400 ppm, 10 min) or glutaraldehyde (40 ppm, 5 min). Disinfection promoted microbial diversity and enrichment of beneficial taxa, highlighting a microbiota programming effect linked to improved larval outcomes. Our results do not support the use of iodophor, even though a particular concentration (20 ppm, 10 min) improved hatching rates, given the observed metagenomic stress indicators and reduced survival at 10 DPH. For hatchery application, our study establishes species-specific, environmentally informed disinfection protocols for *T. blochii*, specifying optimal concentration, exposure time, and frequency and warrants the need for similar investigations in other aquaculture-relevant species. Post-disinfection probiotic supplementation may also further support larval health. Together, these strategies provide actionable guidance for improving survival and overall hatchery performance. Future studies should validate these approaches across species and investigate the mechanistic links between early microbial colonisers and host immunity, paving the way for microbiome-informed hatchery practices.

## Supplementary Information

Below is the link to the electronic supplementary material.

Supplementary Material 1

Supplementary Material 2

Supplementary Material 3

Supplementary Material 4

Supplementary Material 5

## Data Availability

Metagenomic sequencing data were placed in the NCBI-SRA (Sequence Read Archive) database under Bioproject PRJNA765138 (Accession numbers: SRR34022291 to SRR34022302).

## References

[1] Anil, M. et al. National broodbank serving the marine finfishfarming sector of India of Pompano. *Aquacult. Spect.* **4** (9), 29–42 (2021).

[2] Wang, M. et al. The programming of antioxidant capacity, immunity, and lipid metabolism in dojo loach (*Misgurnus anguillicaudatus*) larvae linked to sodium chloride and hydrogen peroxide pre-treatment during egg hatching. *Front. Physiol.* 12. 10.3389/fphys.2021.768907 (2021). 10.3389/fphys.2021.768907 PMC8581469 34777025

[3] Lahnsteiner, F. & Kletzl, M. On-feeding and juvenile production of coregonid species with formulated dry feeds: effects on fish viability and digestive enzymes. *J. Agric. Sci.* **7** (11). 10.5539/jas.v7n11p48 (2015).

[4] Sumithra, T. G. et al. Mechanistic insights into the early life stage microbiota of silver Pompano (*Trachinotus blochii*). *Front. Microbiol.* 15. 10.3389/fmicb.2024.1356828 (2024). 10.3389/fmicb.2024.1356828 PMC11061439 38694807

[5] Sullam, K. E. et al. Environmental and ecological factors that shape the gut bacterial communities of fish: a meta-analysis. *Mol. Ecol.* **21** (13), 3363–3378. 10.1111/j.1365-294X.2012.05552.x (2012). 22486918 10.1111/j.1365-294X.2012.05552.x PMC3882143

[6] Escaffre, A. M., Bazin, D. & Bergot, P. Disinfection of *Sparus aurata* eggs with glutaraldehyde. *Aquacult. Int.* **9** (5), 451–458. 10.1023/A:1020538701557 (2001).

[7] Rasowo, J., Okoth, O. E. & Ngugi, C. C. Effects of formaldehyde, sodium chloride, potassium permanganate and hydrogen peroxide on hatch rate of African catfish *Clarias Gariepinus* eggs. *Aquaculture* **269** (1–4), 271–277. 10.1016/j.aquaculture.2007.04.087 (2007).

[8] Jia, R. et al. Chronic exposure of hydrogen peroxide alters redox state, apoptosis and Endoplasmic reticulum stress in common carp (*Cyprinus carpio*). *Aquat. Toxicol.* **229**, 105657. 10.1016/j.aquatox.2020.105657 (2020). 33075616 10.1016/j.aquatox.2020.105657

[9] Sinha, A. K., Romano, N., Shrivastava, J., Monico, J. & Bishop, W. M. Oxidative stress, histopathological alterations and anti-oxidant capacity in different tissues of largemouth bass (*Micropterus salmoides*) exposed to a newly developed sodium carbonate peroxyhydrate granular algaecide formulated with hydrogen peroxide. *Aquat. Toxicol.* **218**, 105348. 10.1016/j.aquatox.2019.105348 (2020). 31812647 10.1016/j.aquatox.2019.105348

[10] Jia, R. et al. Immune, inflammatory, autophagic and DNA damage responses to long-term H_2_O_2_ exposure in different tissues of common carp (*Cyprinus carpio*). *Sci. Total Environ.* **757**, 143831. 10.1016/j.scitotenv.2020.143831 (2021). 33248772 10.1016/j.scitotenv.2020.143831

[11] Percie du Sert, N. et al. The ARRIVE guidelines 2.0: updated guidelines for reporting animal research. *PLoS Biol.* **18** (7), e3000410. 10.1371/journal.pbio.3000410 (2020). 32663219 10.1371/journal.pbio.3000410 PMC7360023

[12] Animals Scientific Procedures, U. K. & Act U.K. Animals Scientific Procedures Act. (1986). https://www.legislation.gov.uk/ukpga/1986/14/contents

[13] EU Directive 2010/63/EU for animal experiments. Legislation for the protection of animals used for scientific purposes. (2019). https://ec.europa.eu/environment/chemicals/lab_animals/legislation_en.htm

[14] Brown, M. R., Jeffrey, S. W., Volkman, J. K. & Dunstan, G. A. Nutritional properties of microalgae for mariculture. *Aquaculture* **151** (1–4), 315–331 (1997).

[15] Camacho-Rodríguez, J. et al. A quantitative study of eicosapentaenoic acid (EPA) production by *Nannochloropsis Gaditana* for aquaculture as a function of Dilution rate, temperature and average irradiance. *Appl. Microbiol. Biotechnol.* **98** (6), 2429–2440 (2014). 24318007 10.1007/s00253-013-5413-9

[16] Amritha, J. et al. Profiling the antioxidant biomarkers in marine fish larvae: a comparative assessment of different storage conditions to select the optimal strategy. *Fish Physiol. Biochem.* **50** (2), 557–574. 10.1007/s10695-023-01290-6 (2024). 38193995 10.1007/s10695-023-01290-6

[17] Sumithra, T. G. et al. Comparative evaluation of fish larval preservation methods on Microbiome profiles to aid in metagenomics research. *Appl. Microbiol. Biotechnol.* **106** (12), 4719–4735. 10.1007/s00253-022-12026-6 (2022a). 35739345 10.1007/s00253-022-12026-6

[18] Weber, N. et al. Nephele: a cloud platform for simplified, standardized and reproducible microbiome data analysis. *Bioinformatics (Oxford England)*, **34** (8), 1411–1413. 10.1093/bioinformatics/btx617 (2018). 10.1093/bioinformatics/btx617 PMC5905584 29028892

[19] Hammer, Ø., Harper, D. A. T. & Paul, D. R. PAST: paleontological statistics software package for education and data analysis. *Palaeontologia Electronica*, **4**,(1) 9. (2001).

[20] Mandal, S. et al. Analysis of composition of microbiomes: a novel method for studying microbial composition. *Microb. Ecol. Health Disease*. 26. 10.3402/mehd.v26.27663 (2015). 10.3402/mehd.v26.27663 PMC4450248 26028277

[21] Douglas, G. M. et al. PICRUSt2 for prediction of metagenome functions. *Nature Biotechnolog*y, 38 (6), 685–688. (2020). 10.1038/s41587-020-0548-6 10.1038/s41587-020-0548-6 PMC7365738 32483366

[22] Kanehisa, M., Furumichi, M., Sato, Y., Matsuura, Y. & Ishiguro-Watanabe, M. KEGG: biological systems database as a model of the real world. *Nucleic Acids Res. 53*. D672–D677. 10.1093/nar/gkae909 (2025). 10.1093/nar/gkae909 PMC11701520 39417505

[23] Kanehisa, M. Toward Understanding the origin and evolution of cellular organisms. *Protein Sci.* **28**, 1947–1951. 10.1002/pro.3715 (2019). 31441146 10.1002/pro.3715 PMC6798127

[24] Kanehisa, M. & Goto, S. KEGG: Kyoto encyclopedia of genes and genomes. *Nucleic Acids Res.* **28**, 27–30. 10.1093/nar/28.1.27 (2000). 10592173 10.1093/nar/28.1.27 PMC102409

[25] Wickham, H. et al. Welcome to the tidyverse. *J. open. Source Softw.* **4** (43), 1686 (2019).

[26] Sumithra, T. G. et al. Gut microbes of a high-value marine fish, snubnose Pompano (*Trachinotus blochii*) are resilient to therapeutic dosing of Oxytetracycline. *Sci. Rep.* **14**, 27949 (2024b). 39543167 10.1038/s41598-024-75319-y PMC11564560

[27] Sipos, M. J., Lipscomb, T. N., Wood, A. L., Ramee, S. W. & DiMaggio, M. A. Evaluation of three embryo disinfectants on hatching success in four freshwater ornamental fish species. *North. Am. J. Aquaculture*. **82** (1), 63–70. 10.1002/naaq.10118 (2020).

[28] Salvesen, I. & Vadstein, O. Surface disinfection of eggs from marine fish: evaluation of four chemicals. *Aquacult. Int.* **3** (3), 155–171. 10.1007/BF00118098 (1995).

[29] Torgersen, Y. & Hastein, T. Disinfection in aquaculture. *Revue Scientifique Et Technique De l’OIE*. **14** (2), 419–434. 10.20506/rst.14.2.845 (1995). 7579640 10.20506/rst.14.2.845

[30] Amend, D. F. Comparative toxicity of two iodophors to rainbow trout eggs. *Trans. Am. Fish. Soc.* **103**, 73–78 (1974).

[31] Alderman, D. J. The toxicity of iodophors to salmonid eggs. *Aquaculture* **40**, 7–16 (1984).

[32] Kakurinov, V. Food Safety Assurance Systems: Cleaning and Disinfection, Editor(s): Yasmine Motarjemi). *In*: Encyclopedia of Food Safety, Academic Press, pp: 211–225, (2014). 10.1016/B978-0-12-378612-8.00356-5

[33] World organisation for animal health. Recommendations for surface disinfection of salmonid eggs. OIE - Aquatic Animal Health Code – 2/07/2024. (2024). http://chrome-extension://efaidnbmnnnibpcajpcglclefindmkaj/https://www.woah.org/fileadmin/Home/eng/Health_standards/aahc/current/chapitre_disinfection_eggs.pdf (Accessed on 10/12/2024).

[34] McCarty, N., Voorhees, J. M., Barnes, M. E. & Bergmann, D. Different iodine concentrations impact Walleye (*Sander vitreus*) egg survival and the number of bacteria on the chorionic membrane. *Aquaculture J.* **5** (1), 3. 10.3390/aquacj5010003 (2025).

[35] El-Gawad, A., Eman, A., Shen, Z. & Wang, H. Efficacy of formalin, iodine and sodium chloride in improvement of egg hatching rate and fry survival prior to the onset of exogenous feeding in yellow perch. *Aquac. Res.* **47** (8), 2461–2469. 10.1111/are.12694 (2016).

[36] Rach, J. J., Gaikowski, M. P., Howe, G. E. & Schreier, T. M. Evaluation of the toxicity and efficacy of hydrogen peroxide treatments on eggs of warm-and Coolwater fishes. *Aquaculture* **165** (1–2), 11–25 (1998).

[37] Salvesen, I., Oie, G. & Vadstein, O. Surface disinfection of Atlantic halibut and turbot eggs with glutaraldehyde: evaluation of concentrations and contact times. *Aquacult. Int.* **5** (3), 249–258. 10.1023/A:1018343602872 (1997).

[38] Tendencia, E. A. Effect of iodine disinfection on the bacterial flora and hatching rate of *Epinephelus coioides* eggs at the cleavage and eyed stages. *Bull. Eur. Assoc. Fish. Path*. **21** (4), 160–163 (2001).

[39] Wang, M., Hou, J. & Deng, R. Co-exposure of environmental contaminants with unfavorable temperature or humidity/moisture: joint hazards and underlying mechanisms. *Ecotoxicol. Environ. Saf.* **264**, 115432 (2023). 37660530 10.1016/j.ecoenv.2023.115432

[40] Leung, H. W. Ecotoxicology of glutaraldehyde: review of environmental fate and effects studies. *Ecotoxicol. Environ. Saf.* **49** (1), 26–39 (2001). 11386713 10.1006/eesa.2000.2031

[41] Harboe, T., Huse, I. & Øie, G. Effects of egg disinfection on yolk sac and first feeding stages of halibut (*Hippoglossus Hippoglossus L*.) larvae. *Aquaculture* **119** (2–3), 157–165. 10.1016/0044-8486(94)90172-4 (1994).

[42] Jodun, W. A. & Millard, M. J. Effect of iodophor concentration and duration of exposure during water hardening on survival of Atlantic salmon eggs. *North. Am. J. Aquaculture*. **63** (3), 229–233. 10.1577/1548-8454 (2001).

[43] Bögner, D. et al. Hydrogen peroxide oxygenation and disinfection capacity in recirculating aquaculture systems. *Aquacult. Eng.* **92**, 102140 (2021).

[44] Lio, P. A. & Kaye, E. T. Topical antibacterial agents. *Infect. Disease Clin.* **18** (3), 717–733 (2004). 10.1016/j.idc.2004.04.008 15308283

[45] De Swaef, E., Van den Broeck, W., Dierckens, K. & Decostere, A. Disinfection of teleost eggs: a review. *Reviews Aquaculture*. **8** (4), 321–341 (2016).

[46] Attramadal, K. J. K. et al. RAS and microbial maturation as tools for K-selection of microbial communities improve survival in Cod larvae. *Aquaculture* **432**, 483–490. 10.1016/j.aquaculture.2014.05.052 (2014).

[47] Sylvain, F. É. et al. pH drop impacts differentially skin and gut microbiota of the Amazonian fish Tambaqui (*Colossoma macropomum*). *Sci. Rep.* **6** (1), 32032 (2016). 27535789 10.1038/srep32032 PMC4989189

[48] Sumithra, T. G. et al. Metagenomic signatures of transportation stress in the early life stages of Cobia (*Rachycentron canadum*) to aid in mitigation strategies. *Aquaculture* **559**, 738407 (2022b).

[49] Luan, Y. et al. The fish microbiota: research progress and potential applications. *Engineering* **29**, 137–146. 10.1016/j.eng.2022.12.011 (2023).

[50] Kesarcodi-Watson, A., Miner, P., Nicolas, J-L. & Robert, R. Protective effect of four potential probiotics against pathogen-challenge of the larvae of three bivalves: Pacific oyster (*Crassostrea gigas*), flat oyster (*Ostrea edulis*) and scallop (*Pecten maximus*). *Aquaculture* **344-349**, 29–34 (2012).

[51] Wang, L. et al. Effects of thaw slump on soil bacterial communities on the Qinghai-Tibet Plateau, *CATENA*, 232, 107342, (2023). 10.1016/j.catena.2023.107342

[52] Saha, J., Hossain, M. A., Mamun, M. A., Islam, M. R. & Alam, M. S. Effects of carbon-nitrogen ratio manipulation on the growth performance, body composition and immunity of stinging catfish *Heteropneustes fossilis* in a biofloc-based culture system. *Aquaculture Rep.* **25**, 101274 (2022).

[53] Penglase, S. et al. Iodine nutrition and toxicity in Atlantic Cod (*Gadus morhua*) larvae. *PeerJ* **1**, e20. 10.7717/peerj.20 (2013). 23638355 10.7717/peerj.20 PMC3628846

[54] Zhang, R. et al. Povidone iodine exposure alters the immune response and microbiota of the gill and skin in Koi carp. *Cyprinus Carpio Aquaculture*. **563**, 738926 (2023).

[55] Jiao, T. et al. Toxic effects of povidone-iodine on *Macrobrachium rosenbergii*: Concentration-dependent responses in oxidative stress, immunosuppression, and recovery potential. *Animals* **15** (15), 2196. 10.3390/ani15152196 (2025). 40804987 10.3390/ani15152196 PMC12345538

[56] Vadstein, O., Attramadal, K. J., Bakke, I. & Olsen, Y. K-selection as microbial community management strategy: a method for improved viability of larvae in aquaculture. *Front. Microbiol.* **9**, 2730 (2018). 30487782 10.3389/fmicb.2018.02730 PMC6246659

[57] Sun, Y. Q., Wang, J., Shen, C., He, J. Z. & Ge, Y. Plant evenness modulates the effect of plant richness on soil bacterial diversity. *Sci. Total Environ.* **662**, 8–14 (2019). 30682712 10.1016/j.scitotenv.2019.01.211

[58] Thomas, F., Hehemann, J. H., Etienne, R., Mirjam, C. & Gurvan, M. Environmental and gut bacteroidetes: the food connection. *Front. Microbiol.*, **2**(93).10.3389/fmicb.2011.00093 (2011). 10.3389/fmicb.2011.00093 PMC3129010 21747801

[59] Lu, Y. et al. MicrobiomeAnalyst 2.0: comprehensive statistical, functional and integrative analysis of Microbiome data. *Nucleic Acids Res.* **51** (W1), W310–W318. 10.1093/nar/gkad407 (2023). 37166960 10.1093/nar/gkad407 PMC10320150

[60] Zhang, L., Guo, L., Cui, Z. & Ju, F. Exploiting predatory bacteria as biocontrol agents across ecosystems. *Trends Microbiol.* **32** (4), 398–409. 10.1016/j.tim.2023.10.005 (2024). 37951768 10.1016/j.tim.2023.10.005

[61] Rajeev, M. & Cho, J. C. *Rhodobacteraceae* are prevalent and ecologically crucial bacterial members in marine Biofloc aquaculture. *J. Microbiol.* **62** (11), 985–997. 10.1007/s12275-024-00187-0 (2024). 39546167 10.1007/s12275-024-00187-0

[62] Dong, P., Guo, H., Huang, L., Zhang, D. & Wang, K. Glucose addition improves the culture performance of Pacific white shrimp by regulating the assembly of *Rhodobacteraceae* taxa in gut bacterial community. *Aquaculture* **567**, 739254. 10.1016/j.aquaculture.2023.739254 (2023).

[63] Deng, Y., Kokou, F., Eding, E. H. & Verdegem, M. C. J. Impact of early-life rearing history on gut Microbiome succession and performance of nile tilapia. *Anim. Microbiome*. **3** (1), 81. 10.1186/s42523-021-00145-w (2021). 34838149 10.1186/s42523-021-00145-w PMC8627003

[64] Legrand, T. P. et al. The inner workings of the outer surface: skin and gill microbiota as indicators of changing gut health in Yellowtail kingfish. *Front. Microbiol.* **8**, 318469. 10.3389/fmicb.2017.02664 (2018). 10.3389/fmicb.2017.02664 PMC5775239 29379473

[65] Krotman, Y., Yergaliyev, T. M., Shani, A., Avrahami, R., Szitenberg, A. & Y., & Dissecting the factors shaping fish skin microbiomes in a heterogeneous inland water system. *Microbiome* **8** (1), 9. 10.1186/s40168-020-0784-5 (2020). 32005134 10.1186/s40168-020-0784-5 PMC6995075

[66] Dodd, E. T. et al. Influences of claywater and Greenwater on the skin Microbiome of cultured larval Sablefish (*Anoplopoma fimbria*). A*nim. Microbiome*. **2** (27). 10.1186/s42523-020-00045-5 (2020). 10.1186/s42523-020-00045-5 PMC7807797 33499990

[67] Yin, Z. et al. Early life intervention using probiotic *Clostridium Butyricum* improves intestinal development, immune response, and gut microbiota in large yellow croaker (*Larimichthys crocea*) larvae. *Front. Immunol.* **8** (12), 640767. 10.3389/fimmu.2021.640767 (2021). 10.3389/fimmu.2021.640767 PMC7982665 33763082

[68] Gundersen, M. S. et al. Aquaculture rearing systems induce no legacy effects in Atlantic Cod larvae or their rearing water bacterial communities. *Sci. Rep.* **12**, 19812. 10.1038/s41598-022-24149-x (2022). 36396669 10.1038/s41598-022-24149-x PMC9672056

[69] Najafpour, B. et al. Core Microbiome profiles and their modification by environmental, biological, and rearing factors in aquaculture hatcheries. *Mar. Pollut. Bull.* **193**, 115218 (2023). 37441915 10.1016/j.marpolbul.2023.115218

[70] Hanigan, D., Truong, L., Simonich, M., Tanguay, R. & Westerhoff, P. Zebrafish embryo toxicity of 15 chlorinated, brominated, and iodinated disinfection by-products. *J. Environ. Sci.* **58**, 302–310 (2017). 10.1016/j.jes.2017.05.008 28774621

[71] Ding, X. et al. Developmental toxicity of disinfection by-product Monohaloacetamides in embryo-larval stage of zebrafish. *Ecotoxicol. Environ. Saf.* **189**, 110037. 10.1016/j.ecoenv.2019.110037 (2020). 31812018 10.1016/j.ecoenv.2019.110037

