# Supplementary material for: Egg disinfection improves larval survival and shapes the microbial community in snubnose pompano (Trachinotus blochii)
Source: Sci Rep. 2026 Jan 19;16:5761. doi: 10.1038/s41598-026-35646-8 (PMC12894907; doi:10.1038/s41598-026-35646-8)
Supplement: Supplementary file 5 — Supplementary Material 5 [file 41598_2026_35646_MOESM5_ESM.pdf]

Supplementary Files

Supplementary File 1: Abundance data of ASV features at different taxonomic levels in larval microbiota of *T. blochii*

Sheet 1: At phylum level; Sheet 2: At class level; Sheet 3: At order level; Sheet 4: At family level; Sheet 5: At genus level

Supplementary File 2: PICRUSt2 analysis of the whole microbiota profiles of *T. blochii*

Sheet 1: Abundance data of KEGG genes; Sheet 2: Abundance data of KEGG enzymes; Sheet 3: Abundance data of KEGG pathways

Supplementary File 3: ANCOM analysis to find differentially abundant ASVs between different groups

Sheet 1: Differentially abundant ASVs between glutaraldehyde group and control group; Sheet 2: Differentially abundant ASVs between iodophor group and control group; Sheet 3: Differentially abundant ASVs between hydrogen peroxide group and control group; Sheet 4: Differentially abundant ASVs between increased survival group and control group; Sheet 5: Differentially abundant ASVs between increased survival group and iodophor group

Supplementary File 4: Details of ASVs shared between different groups of the present study

Sheet 1: ASVs that are shared across all the study groups; Sheet 2: ASVs present only in the iodophor group; Sheet 3: ASVs present only in the control group; Sheet 4: ASVs present only in group with increased survival; Sheet 5: ASVs that are shared across iodophor and control groups; Sheet 6: ASVs that are shared across iodophor and increased survival groups; Sheet 7: ASVs that are shared across the control and increased survival groups.

Supplementary File 5: Correlations between the ASV features of larval microbiota at the phylum level

Kendall's Tau correlations were shown. Significant positive correlations ($P \leq 0.05$) are indicated by blue bubbles, and negative correlations are indicated by red-coloured bubbles. The size of bubbles indicates the strength of correlation. Nonsignificant correlations were kept as blanks.

Supplementary File 5: Correlations between the ASV features of larval microbiota at phyla level

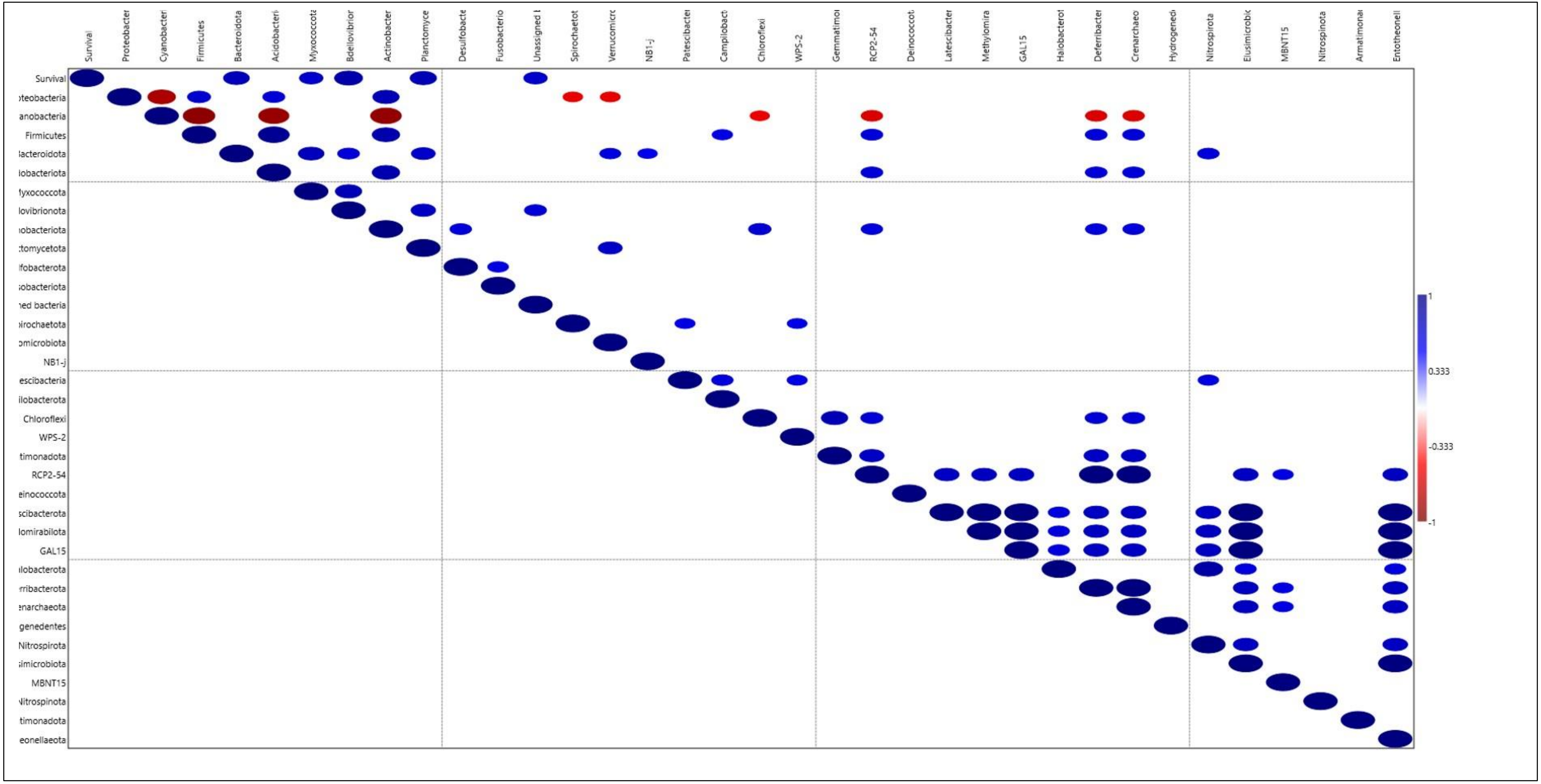

Kendall's Tau correlations were shown. Significant positive correlations ($P \leq 0.05$) are indicated by blue bubbles and negative correlations are indicated by red coloured bubbles. The size of bubbles indicates the strength of correlation. Nonsignificant correlations were kept as blanks.
